# Strategy and Future Prospects to Develop Room-Temperature-Recoverable NO_2_ Gas Sensor Based on Two-Dimensional Molybdenum Disulfide

**DOI:** 10.1007/s40820-020-00558-3

**Published:** 2021-01-04

**Authors:** Abhay V. Agrawal, Naveen Kumar, Mukesh Kumar

**Affiliations:** Functional and Renewable Energy Materials Laboratory, Indian Institute of Technology Ropar, Rupnagar, Punjab 140001 India

**Keywords:** MoS_2_, NO_2_ gas sensors, Light illumination, Heterojunction

## Abstract

Nitrogen dioxide (NO_2_), a hazardous gas with acidic nature, is continuously being liberated in the atmosphere due to human activity. The NO_2_ sensors based on traditional materials have limitations of high-temperature requirements, slow recovery, and performance degradation under harsh environmental conditions. These limitations of traditional materials are forcing the scientific community to discover future alternative NO_2_ sensitive materials. Molybdenum disulfide (MoS_2_) has emerged as a potential candidate for developing next-generation NO_2_ gas sensors. MoS_2_ has a large surface area for NO_2_ molecules adsorption with controllable morphologies, facile integration with other materials and compatibility with internet of things (IoT) devices. The aim of this review is to provide a detailed overview of the fabrication of MoS_2_ chemiresistance sensors in terms of devices (resistor and transistor), layer thickness, morphology control, defect tailoring, heterostructure, metal nanoparticle doping, and through light illumination. Moreover, the experimental and theoretical aspects used in designing MoS_2_-based NO_2_ sensors are also discussed extensively. Finally, the review concludes the challenges and future perspectives to further enhance the gas-sensing performance of MoS_2_. Understanding and addressing these issues are expected to yield the development of highly reliable and industry standard chemiresistance NO_2_ gas sensors for environmental monitoring.

## Highlights

- MoS_2_ shows enormous potential for gas sensing due to its high surface to volume ratio, position-dependent gas molecules adsorption and easy control on morphology.
- The recent experimental and theoretical strategies to develop NO_2_ chemiresistance sensors based on MoS_2_ are addressed.
- A detailed overview of the fabrication of MoS_2_ chemiresistance sensors in terms of devices, structure, morphology, defects, heterostructures, metal doping, and under light illumination are discussed.

## Graphical abstract

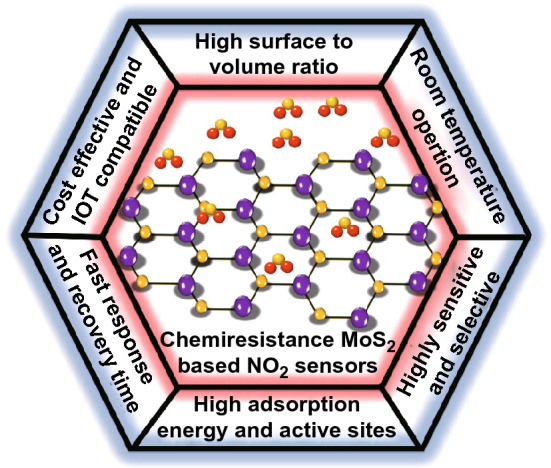

## Introduction

The earth’s environment consists of various chemical elements, gases, and dust particles such as N_2_, O_2_, CO, CO_2_, NO_2_, NH_3_. Among these gases, O_2_, present in the environment is beneficial to living beings, while some gases, such as CO_2_, NO_2_, are toxic and dangerous. The presence of these toxic gases is majorly fixed in the environment. Among all toxic and dangerous gases, NO_2_, a hazardous gas, acidic in nature, highly reactive with a stinky smell is continuously being produced and liberated in the atmosphere due to human activity [1–6]. NO_2_ is produced by fossil fuel burning, forest fires, industry and motor vehicles [7–9]. NO_2_ has recently become a matter of concern in Europe and Australia, owing to its increased concentration. The recent satellite data revealed an unprecedented increase in NO_2_ concentration due to motor vehicles, power plants and wildfire in Europe and Australia in the year 2019 [10–14]. Moreover, after the worldwide outbreak of the novel COVID-19 virus, the lockdown was implemented in highly affected countries, which resulted in the shutdown of factories, manufacturing firms, and transport. This lockdown benefitted the world inadvertently with a dramatic reduction in NO_2_ emissions. Importantly, the high reactiveness of NO_2_ molecules with moisture and its tendency to create an acidic environment makes NO_2_ production thought of concern [5, 15, 16]. It causes respiratory diseases beyond a certain NO_2_ concentration limit in the environment, e.g. coronary assault, cancer, asthma, pneumonia, coughing and bronchitis [16–18]. The presence of NO_2_ in the environment makes the air hazy and thick, which reduces the visibility of human eyes. In addition, the World Health Organization (WHO) reported that major cities around the world had failed to qualify the WHO's air quality standards [19]. An estimated 30.7 million people died due to cardiovascular disease, cancer and chronic respiratory disease in 2016 [19, 20]. Thus, considering the toxicity and hazardousness of NO_2_ gas, there is an urgent need to detect the precise levels of NO_2_ gas in the environment.

A hazardous gas in the environment can be detected by a gas sensor which is an electronic device having two-essential parts; a receptor unit and a transducer unit. Chemical information generated due to gas molecules exposure is gathered and stored in the form of chemical energy in the receptor device. The energy stored in the transducer component is transmuted to an analytical signal [21]. Hulanicki et al. categorized the gas sensors into six classes depending on the transducer mechanism: (1) electrochemical, (2) mass sensitive, (3) magnetic, (4) optical, (5) thermoelectric, and (6) electrical. The classification of gas sensors is carried out on the basis of their transducer operating principle. In today’s fast moving and unstoppable life, the rapid detection of low concentration of toxic gases is indispensable. Among all gas sensors, electrical transducer-based NO_2_ gas sensor has grabbed the prime attention due to their easy handling, simple fabrication process, easy to connect with IOTs, real-time gas detection provision, low-cost and power consumptions, small size and long-term stability in harsh working conditions. In electrical or chemical resistance sensors, the resistance of the sensing material is changed due to charge transfer between the gas molecules and the sensing materials whenever the gas molecules are exposed to the sensing device. The chemiresistance gas sensors have extensive applications in H_2_, NH_3_, NO, H_2_S, NO_2_ gas detection in the environment, industry, cities, space science, transport, vehicles, cultivation, indoors, and various health sectors [22, 23]. Some figures of merits are specified to compare the performance of a gas sensor with different sizes, morphologies and operating conditions, i.e. sensor response, response and recovery time, and selectivity. Generally, sensor response is the ratio of change in resistance with exposure of gas molecules to the resistance of the film before the exposure of gas molecules. It is given by different forms of expression by many groups such as $S = \frac{{\left( {R_{{{\text{gas}}}} - R_{{{\text{air}}}} } \right)}}{{R_{{{\text{air}}}} }}$; $\frac{{\left( {R_{{{\text{air}}}} - R_{{{\text{gas}}}} } \right)}}{{R_{{{\text{air}}}} }}$; $\frac{{\left( {I_{\text{gas}} - I_{\text{air}} } \right)}}{{I_{\text{air}} }};$ $\frac{{R_{\text{gas}} }}{{R_{\text{air}} }}$, $\frac{{R_{\text{air}} }}{{R_{\text{gas}} }}$ [17, 24–36]. Where, $R_{\text{gas}}$($I_{\text{gas}}$) is the resistance (current) of the sensing film in the presence of the gas molecules, $R_{\text{air}}$($I_{\text{air}}$) is the resistance (current) of the sensing film in the presence of the air and S is the sensor response. The response time is the time taken by any gas sensor to attain 90% of the maximum sensor response when the gas is introduced to the sensor. The recovery time is the time taken by any gas sensor to reach 10% of the maximum sensor response when the gas is turned off. The capacity of a gas sensor to respond to a particular gas in the presence of other gases is called selectivity ability of the gas sensor. Usually, sensing films are sensitive to every gas present in the atmosphere at a same time. Also, some gases have nearly same sensor response for specific sensing film. It is therefore difficult to determine the exact change in the sensor response generated by the target gas. Therefore, sensing film must be very selective for the target gas with highest sensor response.

Graphene as a 2D material has some unique properties such as the large surface area (2360 m^2^ g^−1^), zero rest mass of charge carriers near Dirac points and high carrier mobility 200,000 cm^2^ V^−1^ s^−1^ at room temperature (RT) [37–42]. Similarly, other 2D layered materials have numerous properties and applications in comparison to their bulk form [43–45]. The intriguing properties of 2D TMDCs are their high surface to volume ratio, absence of dangling bonds in the pristine form, strong spin–orbit coupling interaction and the high interaction ability for the gas molecules adsorption [46–52]. These features of 2D materials offer interest in exploring their new fundamental physics [32, 53]. The layer-dependent mechanical, electronic, and optical properties of 2D materials create curiosity to learn and explore their fundamental properties [54–56]. A one atom thick layer of graphene has shown an appealing role in gas sensing by detecting 1 ppb concentration of various gases such as NH_3_, NO_2_, H_2_O, and CO [57]. Gas sensors based on graphene have been widely inspected and employed owing to its high carrier mobility, mechanical strengths greater than to steal, remarkable optical and electronic properties [58–60]. Despite having an impressive sensor response and response time, the NO_2_ sensors have suffered from long recovery time owing to the very high adsorption energy of gas molecules with graphene [61–63]. In terms of growth and production, the synthesis of graphene is very costly with the use of toxic chemicals at high temperatures [64–67]. Another challenge associated with graphene is the production of high quality and large surface area graphene film, which is very difficult to attain and the presence of any non-carbon elements disrupts the hexagonality of graphene [68]. Moreover, graphene has zero bandgap, and less environment stability which reduces the gas-sensing performance and long term stability of graphene-based sensors [47, 69].

**Fig. 1:**
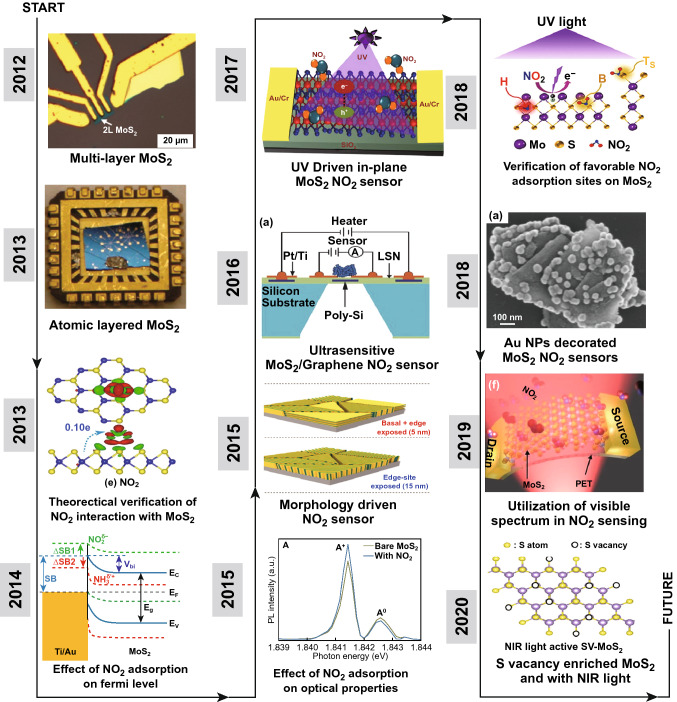
Schematic representation of the 8-year journey of MoS_2_-based NO_2_ sensors. Reproduced with permission from Refs. [34, 118]. Copyright @ Wiley-VCH; Refs. [17, 32, 35, 36, 119, 120, 123]. Copyright @ American Chemical Society; Ref. [117]. Copyright @ Springer; Ref. [121]. Copyright @ AIP Publishing; Ref. [122]. Copyright @ Elsevier

These limitations of graphene mold the direction of research to discover new nonzero bandgap 2D materials like MoS_2_, MoSe_2_, MoTe_2_, WS_2_, WSe_2_, BP, and many more [70–83]. The interaction between the gas molecules and sensing materials is the indelible part of any gas-sensing process. In 2D materials, especially MoS_2,_ is at the forefront in the race of an ideal gas-sensing material [84, 85]. The other substitutes of the 2D materials family are WS_2_, WSe_2_, NbSe_2_, MoTe_2,_ etc. [86–90]. However, most of the research on NO_2_ detection is carried out with MoS_2_. MoS_2_-based gas sensors have achieved noticeable research interest in recent years. MoS_2_ has already shown emerging environmental applications in energy storage, light interaction, flexible electronic devices and in biofield due to its semiconducting nature [50, 91–96]. MoS_2_ has two possible crystal phases, trigonal and hexagonal, where hexagonal is semiconducting while trigonal is having metallic nature [97]. The presence of weak Van der Waals force enables the easy isolation of layers from bulk MoS_2_. The indirect bandgap of 1.2 eV in bulk MoS_2_ is converted to a direct bandgap of 1.8 eV for monolayer MoS_2_ [50, 98, 99]. The absence of dangling bonds provides stability to pristine MoS_2_ flakes in liquid and gaseous media in the presence of oxygen. These facilities make MoS_2_ compatible for gas-sensing application [100]. The low binding energy of 6.1 and 13.9 eV is needed to create S and Mo vacancies, respectively, which can turn the edges of MoS_2_ flakes into metallic sites [101, 102]. MoS_2_ has a tunable bandgap compared to graphene which increases the overall sensing performance of MoS_2_ film [103]. The MoS_2_ flakes have strong photoluminescence (PL) absorption due to the presence of direct bandgap, helpful to design the optical gas sensors. The high on/off ratio (10^8^), the high carrier mobility of 400 cm^2^ V^−1^ s^−1^ at RT, low effective electron mass of 0.48 m_e_ are advantageous for developing fast gas sensors [54, 104–106]. Owing to these electronic properties, any minor change in the electron concentration of MoS_2_ flakes can be easily detected. MoS_2_ flakes have four Raman active modes ($$E_{1g} , E_{2g}^{1} , A_{1g} , E_{2g}^{2}$$). The $$E_{2g}^{1}$$ mode is an in-plane mode and $$ A_{1g}$$ is an out of plane mode [107, 108]. Chakraborty et al. studied in situ Raman spectroscopy of single-layer MoS_2_ flakes [109]. It has been found that $$E_{2g}^{1}$$ is not sensitive to electron doping while the $$ A_{1g}$$ mode is very sensitive to electron doping [109]. With higher electron concentration, the $$A_{1g}$$ mode gets soften due to stronger electron–phonon coupling mode than $$E_{2g}^{1}$$ mode [109]. These vibrational characteristics are ideal for the chemiresistance gas sensors where charge concentration has remained an important parameter. Furthermore, MoS_2_ film has impressive mechanical and optical properties with high Young’s modulus up to 300 GPa, deformity up to 11% without any fracture and amazing transparent nature, making it a potential candidate for optical and flexible devices [110–114]. Moreover, MoS_2_ flakes can be bent up to the radius of 0.75 mm, without deteriorating its electronic properties [115]. Excellent gas molecules detection ability, enormous active sites, large surface to volume ratio and presence of favorable adsorption sites have endorsed MoS_2_ as the unique sensing material. The development and key accomplishment of MoS_2_-based NO_2_ gas sensors in last 8 years are summarized in Fig. 1. With the discovery of the graphene by mechanical exfoliation (ME) technique by the Geim and Novoselov, they further revealed in 2005 that the ME technique can also be employed to thin down the other bulk materials such as MoS_2_ [43]. Following the uniqueness of the MoS_2_, Li et al. developed the NO_x_ sensitive gas sensor [34]. In a similar year, He et al. developed the NO_2_ sensor based on multilayer MoS_2_ flakes and confirm the role of MoS_2_ in NO_2_ detection [42]. The fundamental research to study the electronic properties of MoS_2_ was boosted after the fabrication of first MoS_2_ transistor by Kis et al. [116]. In 2013, Late et al. studied the role of negative and positive back gate voltage on NO_2_ sensing by fabricating the MoS_2_ field effect transistor (FET) [17]. The year 2014–2015 was devoted to the charge transfer mechanism due to the gas molecules exposure. Yue et al. investigated theoretically and confirmed that gas molecule detection in MoS_2_ is attributed to the charge transfer process [117]. Liu et al. demonstrated that NO_2_ gas adsorption strongly affects the Schottky barrier height (SBH) [36]. Cho et al. performed the in-situ PL spectroscopy and investigated the p-type doping in MoS_2_ flakes due to NO_2_ exposure [32].

Till 2015, MoS_2_ has been established itself as the potential candidate for the gas sensing with a well-defined gas-sensing mechanism. However, MoS_2_-based NO_2_ sensors suffered from the incomplete recovery due to the high adsorption of NO_2_ on MoS_2_. Cho et al. studied the role of active sites in gas sensing [35]. NO_2_ adsorption is very high at the active sites in MoS_2_. The active sites are highest at the edges due to presence of dangling bonds, defects and vacancies, while the terrace of MoS_2_ is inert due to absence of dangling bonds. Authors synthesized MoS_2_ flakes of three different orientations: in-plane MoS_2_, mixed MoS_2_ and vertical aligned MoS_2_ flakes. The number of active sites and NO_2_ sensing performance were highest in the case of vertical MoS_2_ flakes. Several studies have been published in parallel years for the fabrication of hybrid MoS_2_ heterostructures to improve the charge transfer in MoS_2_. Long et al. fabricated the low temperature MoS_2_/graphene hybrid structure and develop ultrasensitive NO_2_ sensors up to 50 ppb [118]. Although researchers have achieved full recovery at high temperatures, but the production of RT-recoverable gas sensors has remained a challenging task.

Since 2017, light-assisted NO_2_ sensors have attracted the worldwide scientific community. Rahul et al. in 2017, investigated the role of ultraviolet (UV) light in basal plane MoS_2_ flakes and achieved the full recovery at RT under UV light illumination. Agrawal et al. demonstrated the role of favorable NO_2_ adsorption sites in MoS_2_ by synthesizing the unique morphology of MoS_2_ flakes [119, 120]. Metal NP doping has theoretically proven to be a great combination for enhanced gas sensor response, reactivity and recovery in the past years. Zhou et al. developed the MoS_2_ sensor decorated with Au NPs [121]. It is important to remember that, until 2018, most of the published report used only UV light to boost the efficiency of the sensing light. In the next years, 2019 and 2020 (running) researchers fabricated the visible spectra and near infrared (NIR) spectra-driven NO_2_ sensors [122, 123].

Thus we may conclude that gas-sensing characteristics of MoS_2_ film-based device are highly dependent on size, shape, thickness, morphology, growth direction, polytype composition, defects, metal functionality and the hybrid structure of MoS_2_ films. These factors can be used to classify MoS_2_-based NO_2_ sensors [25, 42, 124].

Apart from the experimental efforts, theoretical studies have also played a noticeable role in designing the experiments and predicting the gas-sensing potential of the proposed materials [125]. Theoretical methods such as density functional theory (DFT) always prove their advantage in terms of time, efforts and cost [125]. DFT provides a broad and detailed view to understand the fundamental mechanism happening between the gas molecules and the sensing material [126, 127]. The key features of DFT are the pre-calculation of the charge transfer and understanding of fundamental interaction between the sensing material and gas molecules. These features are helpful to understand the physical and chemical adsorption of gas molecules, theoretical estimation of defects, their effects on electronic and optical properties and functionalizing the defects with other materials and noble metals. Very few reviews are focused on both the theoretical contribution and the experimental contribution of MoS_2_ for NO_2_ sensing.

The goal of this review is to discuss in detail the MoS_2_-based NO_2_ gas sensors and to provide in-depth insights into previously established theoretical and experimental approaches. We focused on the various properties of MoS_2_ which played a vital role in gas sensing. Mainly, the role of 1T and 2H MoS_2_ phases, large surface area available in MoS_2_ film for gas molecule adsorption, faster charge transport in MoS_2_, effect of modulating favourable adsorption sites via morphology, optical properties and defects available in MoS_2_ will be discussed.

**Fig. 2:**
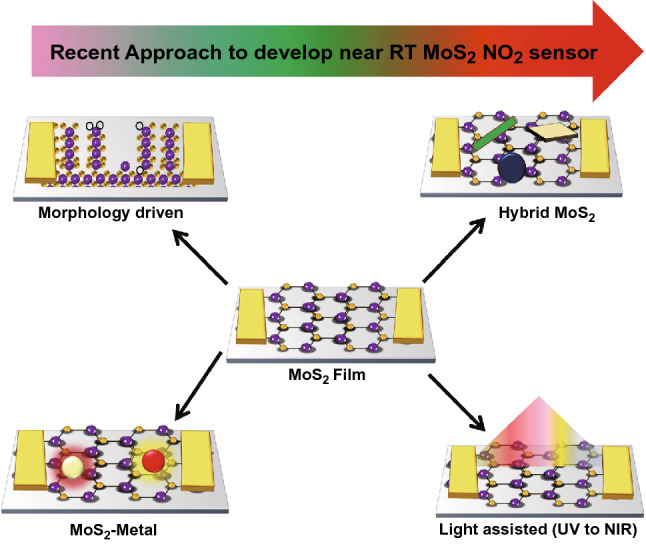
Schematic representation of strategies adopted to develop a high-performance NO_2_ gas sensor based on MoS_2_ flakes

Considering all these points, we have categorized various strategies for enhancing the performances of MoS_2_ sensors as follows: role of device structure (resistor and transistor), monolayer MoS_2_, multilayer MoS_2_, defect tailoring, morphology engineering, heterostructures, functionalizing with noble metals and light-assisted NO_2_ sensors. We have focused our present review in the direction as mentioned above and a schematic view is shown in Fig. 2.

We also focus a little bit on the traditional NO_2_ sensing materials such as metal oxides and carbon-based nanomaterials to gain a clear difference between NO_2_ sensing performance of traditional materials and MoS_2_.

A tremendous effort has been employed to develop fast, high sensor response, selective and low-cost NO_2_ electrical sensors. Various nanomaterial-based sensors from zero dimension (0D, quantum dots) [128–135] to two dimensions (2D, metal oxides, TMDCs) [27, 81, 83, 136–138] showed their exceptional detection ability to detect parts per billion (ppb) NO_2_ gas traces [139–143]. Every nanomaterials has its own merits and demerits in the NO_2_ gas detection. The traditional metal oxides (ZnO, SnO_2_, TiO_2_, In_2_O_3_, WO_3_ etc.) based NO_2_ sensors showed a fast response and high sensor response. However, the highly sensitive nature of metal oxides to humidity reduces the sensor response and stability of gas sensors. Moreover, for accelerating the interaction between the gas molecules and metal oxides, metal oxides gas sensors are need to operate at a higher temperature (250–500 °C). High temperature results in the agglomeration of nanomaterials and increase the grain size of the metal oxide film [28, 143–155]. On the contrary, the carbon material-based NO_2_ sensors provide the high sensor response but at RT the desorption rate of gas molecules is too slow. Thus, the CNT-based NO_2_ sensors are suffered from long recovery time [30, 156–158]. In summary, metal oxide and carbon-based NO_2_ sensors are suffered from thermal safety due to high temperature, structure complexity and complex device fabrication, which restricts the use of metal oxides in smart, wearable and next-generation device for the internet of things (IoT).

**Fig. 3:**
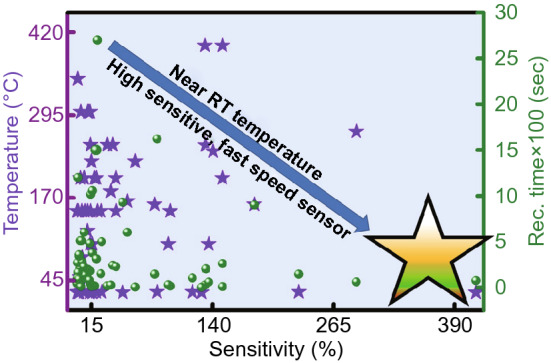
Traditional material-based NO_2_ gas sensors. Most of the traditional NO_2_ sensors have a high operating temperature requirement. The colored star area shows the ideal states for a gas sensor. Data has been taken from Refs. [26, 91, 154, 160–209]

The problems associated with metal oxide and carbon-based NO_2_ sensor have demanded the development of new noble materials with advanced gassensing properties. In Fig. 3, we have summarized the NO_2_ detection performance of various reported traditional materials-based sensors such as ZnO, SnO_2_, CNTs, TiO_2_, In_2_O_3_ SnS_2_, and WO_3_, in terms of operating temperature, sensor response and recovery time [26, 91, 154, 159–197]. Most of the traditional nanomaterial-based NO_2_ sensors reported good sensor response at high operating temperatures (purple star) and simultaneously, they also suffered from the high recovery time (green circles). However, for an ideal gas sensor, it should be operated near RT for high sensing performances. The ideal sensor should have a high sensor response, lower response, and recovery time near to RT, as shown in star region of Fig. 3. Therefore, there is a great demand to develop a low temperature, highly sensitive and fast NO_2_ sensors.

The roadmap of the review is as follows. In Sect. 1, we introduced MoS_2_ as the NO_2_ sensors and addressed its benefits over the traditional metal oxide sensors. In Sect. 2, we will present some peculiar properties of MoS_2,_ which played a critical role in gas molecule adsorption. Section 3 is focused on the interaction mechanism of NO_2_ with MoS_2_ and effect of NO_2_ on electronic, optical and surface properties. In Sect. 4, we will discuss several theoretical findings in which, interaction between NO_2_ and MoS_2_ is discussed. Section 5 covers the experimental reports where bare MoS_2_, morphology-driven MoS_2_, metal-doped MoS_2_, vacancy-driven and photon-assisted MoS_2_-based NO_2_ sensors will be discussed briefly. In Sect. 6, we are going to present some findings where MoS_2_-based heterostructures are utilized for NO_2_ sensing. Finally, in Sect. 7, we will conclude our review and discussed the future of MoS_2_-based NO_2_ sensor.

## MoS_2_: A Unique Material for Gas Sensing

### Structure of MoS_2_

**Fig. 4:**
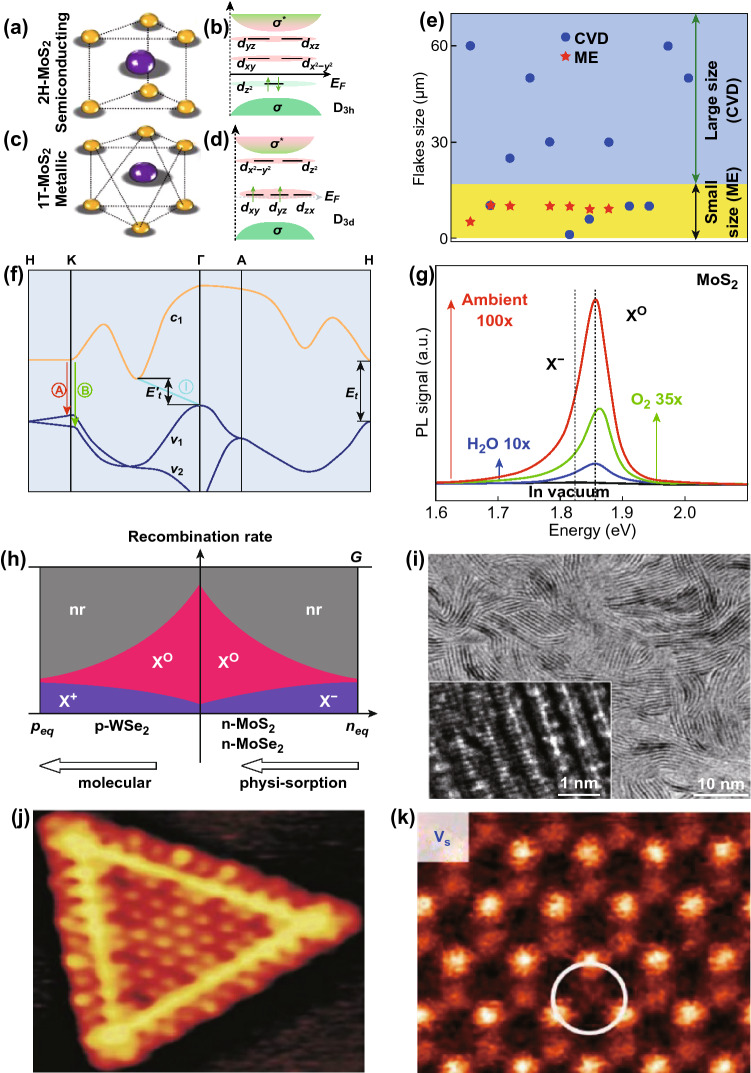
**a** Schematic structure of 2H-MoS_2_. **b** *d*-orbital filling of the semiconducting 2H-MoS_2_. **c** Schematic structure of 1T-MoS_2_. **d** *d*-orbital filling of the semiconducting 1T-MoS_2_. **e** The reported domain size of individual monolayer MoS_2_ flakes from the ME and CVD technique. CVD provides a larger flake size compared to the ME technique. The data of MoS_2_ flakes size has been taken from Refs. [50, 107, 220, 222–239]. **f** Band structure of MoS_2_. The ‘A’ and ‘B’ PL peaks are corresponding to the direct bandgap (*E*_g_) transition of MoS_2_. Reproduced with permission from Ref. [50]. Copyright (2010) American Physical Society. **g** Spectral change in PL due to exposure of O_2_ alone, H_2_O alone and with both. O_2_ and H_2_O incorporate p-type doping which contribute to a blue shift in the peaks. **h** Rate of recombination with neutral exciton and charge trion as a function of charge density in n-type MoS_2_ and p-type MoSe_2_. Reproduced with permission from Ref. [219]. Copyright (2013) American Chemical Society. **i** TEM image of the as grown vertical aligned MoS_2_ flakes. The edges have high catalytic activity than the basal plane and enhance the reactivity of the gas molecules. Reproduced with permission from Ref. [240]. Copyright (2013) American Chemical Society. **j** STM image of the triangular MoS_2_ flakes where yellow perimeters were showing the presence of the metallic states at the edges. Reproduced with permission from Ref. [241]. Copyright (2001) American Physical Society. **k** ADF images of monovacancy S intrinsic defects. Reproduced with permission from Ref. [242]. Copyright (2013) American Chemical Society

The single layer of MoS_2_ has two polymorphs: trigonal prismatic (2H-MoS_2_ Phase) and octahedral phase (1T-MoS_2_ Phase), belonging to *D*_3h_ and *D*_3d_ point groups, respectively. Both polytype structures are shown in Fig. 4a, c [210]. Here, H and T depict hexagonal and trigonal symmetry, respectively, while digits equate to layers repeat per unit cells. In general, the 2H phase is obtained by synthesizing MoS_2_ film using methods such as mechanical exfoliation (ME), chemical vapor deposition (CVD) or ultrasonication [108, 211]. The 1T phase is preferred by the Li intercalation method. The 2H and 1T phases has been widely studied experimentally and theoretically. The 2H-MoS_2_ phase is semiconducting, while the 1T-MoS_2_ phase exhibits metallic nature. The varied electronic nature of MoS_2_ can be understood using crystal field theory (CFT). In CFT, five d orbital $$ d_{{x^{2} - y^{2} }}$$, $$d_{{z^{2} }}$$, $$d_{xy}$$, $$d_{yz} \,{\text{and}}\,d_{zx}$$ of transition metal (Mo) are non-degenerate. These d-bands are located between the bonding ($$\sigma$$) and antibonding bands ($$\sigma^{*}$$), shown in Fig. 4b, d. In trigonal prismatic (*D*_3h_), the orbitals splits into three levels, $$d_{{z^{2} }}$$ ($$a_{1}$$), $$d_{{x^{2} - y^{2} }}$$ + $$d_{xy}$$ ($$e$$) and $$d_{yz} + d_{zx} { }$$ ($$e^{\prime}$$). The octahedral group divided into levels $$e_{g}$$ having $$d_{{z^{2} }}$$ and $$d_{{x^{2} - y^{2} }}$$ orbital and in $$t_{2g}$$ having $$d_{xy}$$, $$d_{yz} \,{\text{and}}\,d_{zx}$$ [212]. When the highest orbitals are partially filled the MoS_2_ possess the metallic like conductivity (1T-MoS_2_, Fig. 4d) and if the highest orbitals are fully filled, MoS_2_ behave like semiconductor (2H-MoS_2_, Fig. 4b). In recent years, a lot of research work has been done on 2H-MoS_2_ phases in gas-sensing applications and many of them addressed in the next sections [17, 34, 35, 42, 120, 213, 214]. The 1T-MoS_2_ has higher active sites and electronic conductivity reaches up to sixfold higher than the 2H-MoS_2_ [99]. Mark et al. prepared a stable metallic phase of MoS_2_ and they observed an enhanced catalytic performance in 1T phase [215]. In addition, the metallic MoS_2_ showed enhanced photoluminescence due to higher sulfur vacancies [99]. Furthermore, Kappera et al. studied the device performance of both phases and observed the low contact resistance at zero bias gate voltage. The low contact resistance generates high drive current with high mobility of 50 cm^2^ V^−1^ s^−1^ [216, 217]. These all properties showed that 1T-MoS_2_ is an important phase for NO_2_ gas sensing. Thus, consideration of the role of both phases in NO_2_ sensing is equally important.

### Large Surface Area for Gas Molecule Adsorption

In contrast to metal oxides, the MoS_2_ has a large specific surface area. The large surface area provides maximum adsorption sites for the adsorption of gas molecules and enhances the surface perturbation in the presence of gas molecules. Moreover, in chemiresistance gas sensors, sensor response is directly proportional to the change in the resistance arises due to the adsorption of gas molecules on the surface [42, 218]. Tongay et al. proposed that if one O_2_ molecule gets physiosorbed on the unit cell of MoS_2_, it withdraws 0.04e per unit cell and the sheet charge density reduced up to 5 × 10^13^ cm^−2^ [219]. Therefore, MoS_2_ is very sensitive and amenable to be used in gas-sensing devices. In this context, MoS_2_ established himself as the promising chemical sensing material due to large highly sensitive surface. CVD, ME, and hydrothermal methods are the most popular methods for synthesizing MoS_2_ for the gas-sensing devices. Among them, the most effective and occupied method to grow large size wafer-scale MoS_2_ flakes is the CVD. We have prepared a comparative graph of flakes sizes with the two most prominent methods i.e., ME and CVD. It has been observed that individual flakes size grown by the ME method can go maximum up to 10 µm. However, with CVD, MoS_2_ flakes of larger size can be grown in comparison with ME. It is worth to mention, we collected data of domain size of only individual MoS_2_ single-layer flakes generated by ME and CVD methods for the data in Fig. 4e. CVD can grow highly uniform, high density, large area and control on morphology of the film while the ME can synthesize highly pure MoS_2_ flakes, which is desirable for many electrical and optical applications. Agrawal et al. synthesized uniform vertical MoS_2_ flakes of 1 × 2 cm^2^ size on SiO_2_/Si substrate. Furthermore, Lin et al. synthesized large size MoS_2_ flakes of 308 µm [220]. Zhan et al. synthesized the centimeter size MoS_2_ layer by CVD method [221]. The nucleation rate, supply of precursors, S and MoO_3_ powder, temperature and the carrier gas flow rate, by CVD is mainly responsible for large area MoS_2_ growth [221, 222]. CVD provides great control on the nucleation rate and mass transport. MoS_2_ flakes size is increased with time as the more and more nucleation center and sites grow over substrate. Hence, CVD is the better option to grow the large area MoS_2_ flakes and to fabricate the gas-sensing devices.

### Impact of Gas Adsorption on Optical Properties of MoS_2_

The photoluminescence (PL) is an essential characterizations to detect changed in the electron concentration of a 2D material-based gas sensor. Gas-sensing ability of 2D materials is governed by either electron depletion or accumulation that depend on the doping behavior of the exposed gas molecules.

The nature of dopants critically affect the PL spectra of MoS_2_. MoS_2_ has two well-reported PL peaks ‘A’ and ‘B’. These PL peaks are emerged due to the splitting of the valence band in $v_{1}$ and $v_{2}$ [50]. The valence band splitting at the K-point is the collective effect of interlayer spacing and spin–orbit coupling. Figure 4f displays the direct bandgap transition peaks (A and B) and indirect bandgap transition (I) in the MoS_2_ crystal structure. The spectral weight of exciton and trions can be significantly tuned by the electrical gating, n-type or p-type molecular adsorption (e.g. H_2_O, TCNQ) doping, and defects present at the cracks [219, 243–245].

Nan et al. studied the role of molecular adsorption on the PL through oxygen exposure [246]. Micro PL analysis revealed the enhancement in PL intensity due to molecules adsorption by MoS_2_ surface at moderate temperatures in high vacuum ambient. The PL spectroscopy was performed over the as prepared monolayer MoS_2_ films, which were annealed for 1 h in vacuum at 350 and 500 °C. It was observed that the PL intensity was increased sixfold after annealing at 350 °C with the blue shift in energy (from 1.79 to 1.81 eV). Moreover, the PL intensity in sample annealed at 500 °C was erratic at different locations. When the MoS_2_ film was annealed at 350 °C, the MoS_2_ film was uniform and environmental O_2_ and H_2_O physically got adsorbed by MoS_2_ flakes. Both O_2_ and H_2_O introduced p-type doping in MoS_2_. When the flakes annealed at 500 °C, cracks were formed in the film with the generation of defects. At these defects’ sites O_2_ and H_2_O adsorbed chemically and introduced heavy *p* doping. DFT calculations were also performed and calculated charge transfer between the O_2_ and pristine MoS_2_ was 0.021e, while at the defects site, the charge transfer was 0.997e. Thus, higher charge transfers at the defective sites introduced heavy p-type doping. Tongay et al. also studied the modulation in the PL due to the physisorption of O_2_ and H_2_O molecules. Physiosorbed O_2_ and H_2_O molecules bonded weakly with MoS_2_ but introduced significant p doping. The variation in PL intensity due to exposure of O_2_ alone (green), H_2_O alone (blue) and with both (red) shown in Fig. 4g [247]. The 0.04e and 0.01e times charges were transferred from MoS_2_ to O_2_ and H_2_O molecules, respectively. The O_2_ and H_2_O molecules adsorption modulate the charge concentration in the MoS_2_. The electrons of the n-type MoS_2_ flakes are depleted by both molecules. Here, the focus has been given on the low energy exciton peak which is the combination of the neutral exciton (*X*^0^) and charge trions ($X^{ + }$/$X^{ - }$). In actual, the MoS_2_ layer has high sheet charge density $\left( {n_{\text{eq}} } \right)$. The high $n_{\text{eq}}$, destabilizes the neutral exciton X^0^ due to electrostatic screening between the holes and free electrons while the $X^{ - }$ stabilizes due to high recombination rate of $X^{ - }$ trions [248]. Hence, with high $n_{\text{eq}}$, the overall PL intensity becomes low. With physisorption and chemisorption of molecules such as O_2_ and H_2_O, the $n_{\text{eq}}$ gets reduced. Hence, the number of electrons available in MoS_2_ for trions formation is decreased. Thus, the intensity of $X^{ - }$ decreased and $X^{0}$ enhanced with more stabilization, as can be seen from Fig. 4g, h. Moreover, it has been reported that the electronic gating and molecular doping can dramatically tune the PL [219, 244, 245, 249]. As the gas molecule adsorption introduces n or p doping, the study of PL with adsorption of molecules to MoS_2_ is an important aspect to understand the nature of the gas molecules.

### High Catalytic Nature and Presence of Metallic States

Another essential feature of MoS_2_ is the presence of a large number of active sites for promoting the chemical reactions. Jaramillo et al. identified the active sites on the MoS_2_ through scanning tunneling microscopy (STM) [250]. The MoS_2_ samples were synthesized on the Au substrate and STM imaging was performed in the ultra-high vacuum. The STM measurements confirmed that flat MoS_2_ edges have bright rims which appeared as bright lines along the flakes perimeter. To validate the high activity of the edges, the hydrogen evolution reactions (HER) activity was investigated which also confirmed the high reactivity of edges. Kong et al. synthesized MoS_2_ by the sulfurization of Mo film deposited by e-beam lithography. The tunneling electron microscopy (TEM) image of vertical aligned MoS_2_ flakes is shown in Fig. 4i and in inset. It is evident from these studies that edges have highly active site. Thermodynamically, the growth of in-plane MoS_2_ is highly probable than the edge oriented MoS_2_ flakes. The high activity of the edges boosts the motivation to grow edge-enriched film by forming the various morphology of MoS_2_ nanoflakes such as vertical aligned MoS_2_, MoS_2_ nanowires, MoS_2_ spheres etc. Kim et al. fabricated 2D SnS_2_ and develop NO_2_ sensor by enhancing the active sites [251]. The vertically aligned SnS_2_ showed high NO_2_ reactivity due to the presence of a large number of active sites in comparison to the basal plane SnS_2_. Shim et al. synthesized SiO_2_ nanorods (NRs) and decorated them with MoS_2_ flakes [252]. These SiO_2_ NRs enhanced the catalytic activity of MoS_2_ flakes by exposing more edges of MoS_2_ flakes [251]. Hence, the NO_2_ detection ability of SiO_2_ NRs encapsulated with MoS_2_ is increased. The MoS_2_ surface has maximum number of active sites which enhance the chemical activity of MoS_2_ film [240, 253, 254]. Another important feature of MoS_2_ flakes is the presence of metallic states at the edges [241]. The MoS_2_ edges behaved as the one-dimensional metallic wires and appeared as the bright brim of high conductance, as shown in Fig. 4j. The attention here is given to Mo edges having S dimers. The Mo edges have two metallic wave functions and generate metallic states in MoS_2_. Therefore, the presence of metallic edges will be helpful in the fast transfer of generated electron and holes. The generated charge can be rapidly transferred along the edges in edge-enriched MoS_2_ and will be helpful in developing the fast responsive and recoverable gas sensors [255].

### Impact of Gas Molecules Adsorption on Schottky Barrier Height

The gas-sensing performance of 2D materials based on chemiresistance gas sensors is critically influenced by the metal contacts [256–259]. In 2D materials, the gas molecule adsorption affects the charge concentrations and carrier density. Depending on the nature of the gas molecules, the charge carrier density either increases or reduces and Fermi level of 2D materials is modulated with gas molecule adsorption. The equilibrium Fermi level of metal and semiconductor before and after exposure to the gas molecule will be different due to variation in the charge carrier density in the sensing film. In chemiresistance sensors, the Schottky barrier height between the metal contact and the 2D material surface can alter the surface charge transfer mechanism. Various studies have been reported to understand the role of Schottky barrier height (SBH) and Schottky barrier modulation (SBM) with gas molecule exposure in traditional gas sensors as well as in 2D material-based gas sensors [260, 261]. The band structure of metal and semiconductor can be divided into two regions: (1) alignment of the energy levels of the metal and semiconductor for charge carrier injection and (2) band bending at the space charge region for charge carrier separation [262]. If the metal and semiconductor work functions are $\emptyset_{M}$ and $\emptyset_{S}$ respectively, the SBH determined by the Mott–Schottky rule is given by Eq. (1):

1 $$ \emptyset_{b} = \emptyset_{M } - \chi_{S} $$

Depending on the type of the semiconductor (n-type or p-type), the Schottky or ohmic contact nature of the junction is decided. In 2D materials, ohmic contacts are of great importance due to their low resistance and high charge transfer in terms of high mobility and current on/off ratio [104, 263, 264]. However, the ohmic contacts are not beneficial for gas-sensing point of view. The reason for this is the interaction of gas molecules with sensing film and their effect on the Schottky barrier modulation (SBM) [36, 72]. The importance of the Schottky contact is well established in the metal oxide sensors. Zhou et al. demonstrated the remarkable performance of the ZnO sensors by utilizing the Schottky contact in comparison to the ohmic contact [265]. Similarly, Wei et al. fabricated the ZnO NW-based CO sensor in such a way that one end behaved as the Schottky contact, while the other end behaved as the ohmic contact [266]. Schottky end behaves like a gate terminal and the Schottky barrier height (SBH) was tuned. Nearly 4 times enhanced sensor response with seven times reduce response and recovery time were observed. In all these reports, SBM provides an efficient and enhanced charge transport. Hence, gas-sensing performance is high in the Schottky contacted devices.

### Role of Defects in Gas Molecule Adsorption

In case of MoS_2_, defects can be generated during the synthesis or transfer of MoS_2_ due to synthesis imperfections [267–270]. In addition, these defects are susceptible to ambient environments conditions [271, 272]. Defects can also be created through the irradiations, metal doping and functionalization [273, 274]. Thus, MoS_2_ structures unavoidably have various defects in terms of vacancies, dopants, adsorbates, adatoms, and impurities. On the contrary, the pristine MoS_2_ is assumed to have defect free surfaces. However, the synthesis of defect free MoS_2_ flakes is quite difficult and convoluted. Defects are easily produced during the synthesis process. Defects crucially affect various mechanical, electronic, optical and catalytic properties. Zhou et al. fabricated MoS_2_ and studied the possible structural defects [242]. The authors studied atomic-resolution annular dark field (ADF) images of CVD-grown MoS_2_ flakes. The defects were classified into six types (i) mono-sulfur Vacancies (V_S_), (ii) di-sulfur vacancies (V_S2_), (iii) Mo atom with three nearby sulfur (V_MoS3_), (iv) Mo atom with three di sulfur pairs (V_MoS6_), (v–vi) Antisite defects, Mo atom at S vacancy site (Mo_S2_) and S atom at Mo vacancy site (S2_Mo_). The formation energy of these vacancies is studied in term of S chemical potential. The formation energy plot revealed that mono S vacancies are most probable and need lowest formation energies. The ADF image of S vacancy site is shown in Fig. 4k. These defects could play a crucial role in the gas molecule adsorption. The benefits of defects in graphene have already received great attention [275, 276]. The findings of the reports revealed that the sensing mechanism in pristine and defective graphene is completely different. The defective graphene has higher interaction with gas molecules due to the presence of the defects. Interestingly in MoS_2_, defects can greatly influence the gas-sensing properties [86, 277]. Moreover, doping defects with substitutional impurities atoms can greatly improve the MoS_2_ sensing performances. The effect of dopant and impurities is also well established in graphene. Zhang et al. studied the sensing performance of graphene-doped B, N, Si, Ca, Co and Fe, defective graphene and on pristine graphene [278]. The defective graphene doped with Ca, Co and Fe showed the highest interaction with H_2_S molecules. In metal-doped graphene, mixing of the graphene orbitals and metals orbitals is enhanced with H_2_S orbitals which leads to the strong interaction.

## Charge Transfer Mechanism Between NO_2_ and MoS_2_: Effect on Electronic Properties, Optical Properties, and Metal Contacts/MoS_2_ Interface

In the present section, the nature and effect of NO_2_ gas molecules on electrical conductivity, PL and MoS_2_ band alignment will be addressed. NO_2_ is a secondary product generated from the primary NO source as shown by Eq. (2) [279].

2 $$ 2{\text{NO}} + {\text{O}}_{2} \to 2{\text{NO}}_{2} $$

NO_2_ has the electron acceptor nature and behaves as a strong oxidizing agent due to the unpaired electrons of nitrogen atom. NO_2_ molecules take the electrons from the sensing materials. Generally, a chemiresistance gas sensor has a sensing layer that detects the presence of interacting gas molecules. The electrical and optical properties changes depending on the nature of interacting gas molecules and the type of semiconducting film. The gas molecules that interact can either behave like a reduction gas (electron donor) or an oxidizing gas (electron acceptor). Similarly, the semiconductor film may also have an n-type or a p-type nature.

In the case of TMDCs materials, gas molecules interaction depends on the nature of TMDC film and gas molecules. The interaction of gas molecules with TMDC film is governed via the physisorption or chemisorption process. The physisorption process occurs with pristine TMDC film while the chemisorption process happens with defective TMDC layers and on the defect sites.

In the case of pristine TMDC films, the gas molecules and TMDC films interact through the physisorption process. The gas molecules have weak adsorption energy and long adsorption distance with pristine TMDC film. Moreover, there is a less charge transfer between the gas molecules and TMDC film with an almost unchanged electronic structure. Hence, gas sensors based on pristine TMDC films have fast recovery but with low sensor response. The physisorption-based gas sensing reported in SnS_2_ [175]. The SnS_2_ showed a highly selective nature for NO_2_ molecules due to the physisorption process. Furthermore, the positive binding energy of O_2_ molecules with the SnS_2_ surface indicated high surface resistance for oxygen molecules and supported that NO_2_ sensing response in SnS_2_ was through the physisorption process [175].

**Fig. 5:**
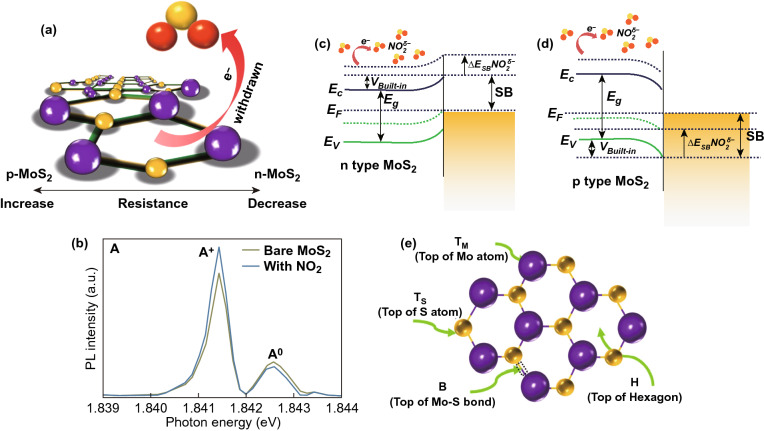
**a** Schematic interaction of NO_2_ gas molecules with the n-type or p-type MoS_2_ layer. NO_2_ captures the electrons from MoS_2_ layer. **b** Effect of NO_2_ molecules adsorption on PL spectra. The spectral weight of positively charged trions is increased on the cost of excitons spectral weight in n-type MoS_2_. Reproduced with permission from Ref. [32]. Copyright (2015) American Chemical Society. Schottky barrier height modulation after NO_2_ molecules adsorption in **c** n-type MoS_2_ **d** p-type MoS_2_. **e** Four possible NO_2_ adsorption sites on MoS_2_. Reproduced with permission from Ref. [255]. Copyright (2017) AIP Publishing

In the case of the chemisorption process, defects induce during the synthesis of MoS_2_. The gas molecules interact chemically with MoS_2_. The chemical interactions of gas molecules enhance gas-sensing performances of sensing material. The adsorption distance between the gas molecules and the adsorption sites is minimal in case of the chemisorption process. Hence, high charge transfer, strong adsorption energy, and significant change in the electronic states have been observed. The charge transfer schematic of NO_2_ with the MoS_2_ film (n-type or p-type) is shown in Fig. 5a and Eq. (3).

3 $$ {\text{NO}}_{2} + {\text{e}}^{ - } \to {\text{NO}}_{2}^{ - } $$

Cho et al. experimentally verified the charge transfer mechanism between the MoS_2_ and NO_2_ gas molecules using PL spectroscopy [32]. The authors synthesized n-type MoS_2_ film by the chemical vapor deposition technique. The authors exposed NO_2_ gas to MoS_2_ film and investigated the charge transfer mechanism using photoluminescence (PL) spectroscopy. The authors observed that with NO_2_ exposure, the resistance of the n-type MoS_2_ film increased (positive sensor response). The increment in the resistance confirmed that NO_2_ withdraws the electrons from the n-type MoS_2_ film. NO_2_ gas molecules exposure modulates the electron concentration in MoS_2_. The change in the electron concentrations dramatically affects the PL. The MoS_2_ has two main PL exciton peaks named as 'A' and 'B' [50]. The intensities of these two PL peaks can either decreased or increased with a change in the electron concentrations [243, 244]. The low energy PL peak 'A' can be expanded into a charged trions ($A^{ + / - }$) and in neutral exciton ($A^{0}$). The MoS_2_ flakes grown on the SiO_2_ substrate showed dominated behavior of $A^{ + }$ peak over $A^{0} .$ Hence, the authors considered the positively charge trion peak $(A^{ + }$) and neutral exciton peak ($A^{0}$). As NO_2_ has an electron acceptor nature, it takes the electron from the MoS_2_ and intensity of the ($A^{ + }$) enhanced due to conversion of neutral exciton in ($A^{ + }$). Actually, the numerous number of holes generated in MoS_2_ due to depletion of electrons by NO_2_. Therefore, intensity of $A^{ + }$ trions enhanced and neutral exciton suppressed. Similar behavior is observed in the PL spectroscopy, shown in Fig. 5b. The effect of NO_2_ exposure on the Fermi level of n-type and p-type MoS_2_ flakes is shown in Fig. 5c, d. MoS_2_ can have both types of semiconducting nature. In both cases, NO_2_ exposure depletes the electrons from the MoS_2_ and manipulate the charge density in the conduction band. Due to electron extraction, the Fermi level in the n-type MoS_2_ film moves downward toward the valence band and correspondingly the SBH and resistance increased. When MoS_2_ film has the p-type nature, holes majority increased with NO_2_ exposure. The Fermi level move toward the conduction band and SBH and resistance decreased. Thus, NO_2_ adsorption critically affects the electronic as well as the optical properties of MoS_2_.

Yue et al. theoretically investigated the adsorption of several molecules using DFT on MoS_2_ such as H_2_, O_2_, H_2_O, NH_3_, NO, NO_2_, and CO [117]. Theoretically, gas adsorption behavior is determined by the few terms namely: favorable adsorption sites on MoS_2_ for particular gas molecule, distance between the gas molecule and the MoS_2_ layer, the binding energy of gas molecule on the MoS_2_ layer, charge transfer between the gas molecules and MoS_2_ layer, and direction of charge transfer. For adsorption of any gas molecule on a sensing surface, there should be a strong favorable interaction between the gas molecules and MoS_2_ flakes, and it should be adsorbed physically or chemically. This interaction is determined in terms of adsorption energy, calculated by Eq. (4):

4 $$ E_{{\text{a}}} = E_{{{\text{MoS}}_{2} + {\text{molecule}}}} - \left( {E_{{{\text{MoS}}_{2} }} + E_{{{\text{molecule}}}} } \right) $$

where $E_{{\text{a}}}$ is the adsorption energy, $E_{{{\text{MoS}}_{2} + {\text{molecule}}}}$ is the total energy of MoS_2_ and the adsorbed gas molecule.$E_{{{\text{MoS}}_{2} }}$ and $E_{{{\text{molecule}}}}$ are the energy of the MoS_2_ film and single gas molecule, respectively. For a strong interaction, the adsorption energy should be negative and the interaction process should be exothermic. Another term is the charge transfer process. The charge transfer process depends on the relative position of the highest occupied molecular orbitals (HOMO) and lowest unoccupied molecular orbitals (LUMO). If the Fermi level is below the HOMO, then charge transfer from molecule to sensing surface and gas is called the electron donor, and if the Fermi level is above the LUMO, then the charge transfer from sensing surface to molecule and gas called is the electron acceptor [280].

As mentioned above, the adsorption of the gas molecule is determined in terms of favorable adsorption sites. The gas molecules adsorption are highly position dependent in the case of MoS_2_ due to the difference in the adsorption energy and charge transfer for gas molecules at different adsorption sites on MoS_2_. The monolayer of MoS_2_ has a hexagonally packed structure where Mo atoms are sandwiched between the two layers of S atoms. There are four possible adsorption sites, the H sites (Top of the hexagon), T_S_ (top of S atom), T_M_ (top of Mo atom), and B (top of Mo and S bond). The possible sites configurations are shown in Fig. 5e. In the case of NO_2_, three different NO_2_ molecules orientations have been considered with these four sites, starting from one N atom with N–O bonds parallel to monolayer, two with NO-bonds pointing up or down to monolayer. After the gas molecule adsorption on MoS_2_, MoS_2_ structure with adsorbed gas molecules is reached to the equilibrium state with the highest adsorption energy.

The minimum distance between the adsorbed gas molecule and the relaxed MoS_2_ surface is called as equilibrium height. The importance of distance between the NO_2_ and top S layer of MoS_2_ is also studied and investigated by Yue et al. The highest adsorption energy was found at an equilibrium height of 2.71 Å. It has to be noted that the highest adsorption energy is negative for adsorption of NO_2_ on MoS_2_, confirming the favorable adsorption of NO_2_ on MoS_2_. Among all, depending on the charge transfer and adsorption energy, the most favorable NO_2_ orientation was estimated. The H, T_S_, and B sites (− 276, − 249, and − 249 meV, respectively) found favorable for NO_2_ adsorption while no adsorption on T_M_ site was observed. The high adsorption energy was attributed to polarization produced in the MoS_2_ sheet during NO_2_ adsorption. Hence, the interaction was determined by the electrostatic force and lead to strong adsorption energy. From the adsorption energy calculations, the highest favorable NO_2_ adsorption site is at the H site. The charge transfer from MoS_2_ to NO_2_ was found to vary from 0.1e to 0.119e. The positive charge transfer value implies the transfer of charge from MoS_2_ to NO_2_. The difference in the charge density due to NO_2_ exposure further confirmed the charge accumulation and depletion profile. The effect of NO_2_ molecule on energy band structure is also studied and it has been found that the adsorbed NO_2_ molecule introduces an unoccupied flat impurity state at 0.31 eV above the Fermi level in the conduction band of MoS_2_. The used method, supercell size, lattice parameters and available favorable adsorption sites for NO_2_ molecules adsorption on MoS_2_ by Yue et al., are tabulated in Table 1. Another important aspect of the work is the study of the applied electric field on the NO_2_ adsorption on MoS_2_. The charge transfer mechanism between the adsorbents and absorber is the key to the gas molecule adsorption.

**Table 1:** Method, supercell size (S.S), lattice parameter (L.P) and favorable adsorption sites on MoS_2_ calculated by Yue et al. using DFT [117]

Method	S. S (L.P)	H-site			T_S_-site			B-site			Reference
		d	*E*_a_	$\Delta Q$	d	*E*_a_	$\Delta Q$	d	*E*_a_	$\Delta Q$	
VASP (LDA)	4 × 4 (3.12)	2.65	− 276	0.1	2.71	− 249	0.119	2.62	− 249	0.114	[117]

The amount of charge transfer is very sensitive to the electric field. The applied electric field is considered in two perpendicular directions (i) MoS_2_ to NO_2_ molecule (+E) and (ii) NO_2_ to MoS_2_ molecule (− E). The charge transfers from MoS_2_ to NO_2_ increase with an increase in the positive electric field and it tends to decrease when the direction of the field is reversed. The negative electric field forces the electrons to transfer from NO_2_ to MoS_2_. The external electric field and dipole moment direction are well correlated with each other. Hence the direction of the electric field is greatly affected by the charge transfer values.

## Theoretical Investigations of NO_2_ Adsorption on MoS_2_

Here, we discuss the reports where the interaction of NO_2_ on MoS_2_, the role of MoS_2_ polytype and metal doping investigated theoretically.

### Adsorption of NO_2_ Gas Molecules on Defective MoS_2_

In the present section, we will discuss some theoretical reports in which adsorption of NO_2_ is studied on the defective MoS_2_. The two types of defects are considered mainly in MoS_2_ the monosulfur vacancies and the Mo-doped S vacancy sites.

**Fig. 6:**
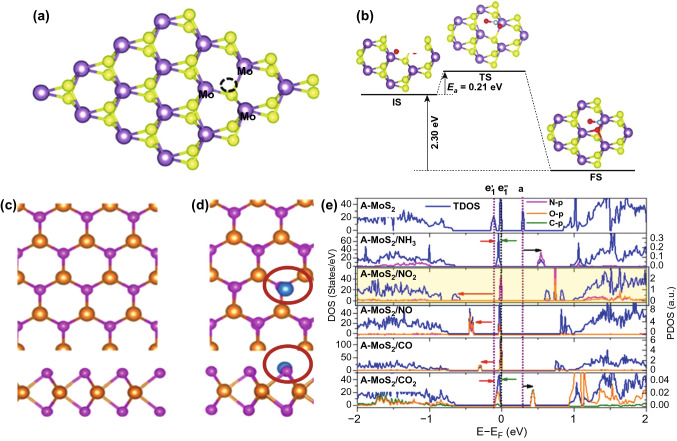
**a** Structure of defected MoS_2_. Black circles represent the S vacancy sites. **b** Dissociation of NO_2_ onto the S vacant MoS_2_. ${\text{Mo}}_{{\text{S}}}$. Reproduced with permission from Ref. [281]. Copyright (2016) The Royal society of chemistry. **c** Top view of pristine MoS_2_ **d** Mo-doped S Antisite defects (Mo_S_) **e** DOS and PDOS of monolayer MoS_2_ with Antisite defect-doped MoS_2_ with gas molecules exposure. Reproduced with permission from Ref. [285]. Copyright (2016) American Chemical Society

Owing to the chemical interaction of NO_2_ with MoS_2_, the adsorption mechanism is governed by the chemisorption mechanism. Li et al. used DFT to study the adsorption of NO_2_ molecule on the single S vacancy site [281]. Initially, the effect of vacancies on the electronic structures was studied. The schematic of a MoS_2_ unit cell is shown in Fig. 6a. The bandgap with a single S vacancy in MoS_2_ was decreased up to 1.07 eV [282, 283]. The S vacancies in 2D materials create midgap states which reduce the bandgap of MoS_2_. These states arise due to the unsaturated Mo atoms near the vacant S sites [284]. When NO_2_ molecules are adsorbed to the MoS_2_ surface, the NO_2_ molecule dissociates at the S vacancy sites into NO and O. The dissociated NO has a bond length equivalent to the free NO molecule. Hence, the dissociated O atom is adsorbed on the single S vacancy site and the other part NO is physiosorbed on the O-doped MoS_2_. The activation barrier energy and transition states were also calculated. The activation energy of NO_2_ dissociation was 0.21 eV and the reaction energy was 2.30 eV, as shown in Fig. 6b. This energetically favored the NO_2_ dissociation process. Furthermore, adsorption energy of physiosorbed NO was 0.44 eV which is minimal and facilitates desorption of NO_2_ molecules from MoS_2_.

Sahoo et al. doped Mo atoms on S vacancy sites, and enhanced adsorption of NO_2_, as shown in Fig. 6c, d with a red circle [285]. This type of doping is termed as antisite defects ($${\text{Mo}}_{S }$$) in MoS_2_ (A-MoS_2_). The insertion of Mo atoms at the S defects sites are highly probable with physical vapor deposition techniques. A-MoS_2_ may be an innovative method to improve the sensor response, selectivity, and sensing performance of the MoS_2_ sensor. The insertion of Mo atom at the S vacancy site generates the three midgap states, two states are at -0.02 and -0.11 eV below the Fermi level and the third state above the Fermi level at 0.28 eV. Actually, 4d orbitals of antisite Mo atom is splitted into three states; a ($$d_{z}^{2}$$) state lies above the fermi level, twofold degenerate $$ e_{1 } (d_{xy} , d_{{x^{2} - y^{2} }}$$), and $$e_{2 } \left( {d_{yz} ,d_{zx} } \right)$$ due to the $$C_{3v}$$ symmetry, lies below the Fermi level. It is worth to note that $$e_{1 }$$ state splits into $$e_{1}^{^{\prime}}$$ and $$e_{1}^{^{\prime\prime}}$$ levels due to the John Teller distortion while $$e_{2 }$$ lies well below the valence band. The corresponding density of states (DOS) and partial density of states (PDOS) of Mo antisite-doped MoS_2_ without NO_2_ and with NO_2_ exposure are shown in Fig. 6e. Finally, when NO_2_ gas molecules are exposed to A-MoS_2_, the NO_2_ interaction process is highly exothermic and higher charge transfer takes place in A-MoS_2_ in comparison to the pristine MoS_2_. The paramagnetic NO_2_ molecules are adsorbed in the tilted configuration. The strong mixing of antisite defect $${\text{Mo}}_{S }$$ and of NO_2_ orbitals are responsible for high charge transfer and strong adsorption energy. The p orbitals of N and O atom of NO_2_ molecules are strongly hybridize with the three new mid gap states generated due to the antisites $${\text{Mo}}_{S }$$ defects. The DOS and PDOS states of A-MoS_2_ confirmed this behavior. The strong hybridization occurred between the NO_2_ molecule and with three new mid gap states which enhanced the charge transfer.

### Adsorption of NO_2_ Gas Molecules on 2H-MoS_2_ and 1T-MoS_2_ Polytype

The two polytype of MoS_2_, 2H-MoS_2_, and 1T-MoS_2_ have their own advantages in NO_2_ sensing. Both polytypes have distinct electronic nature of semiconductors (2H-MoS_2_) and metallic (1T-MoS_2_). Here, in this section, we will enlighten the role of both pristine phases and defective phases MoS_2_ in NO_2_ sensing. Linghu et al. has compared the NO_2_ sensing performance of pristine 2H-MoS_2_ and pristine 1T-MoS_2_ [286]. The 1T-MoS_2_ has shown promising sensing performances in comparison to the 2H-MoS_2_. The geometric optimization revealed that NO_2_ has a closer and stronger interaction with the 1T-MoS_2_ phase than the 2H-MoS_2_. The calculated adsorption energies for the 2H-MoS_2_ and 1T-MoS_2_ phases are -0.21 eV and − 0.25 eV, respectively, reasonable to assume the higher NO_2_ interaction with the 1T phase. The higher adsorption energy comparative to 2H -MoS_2_ confirmed the higher and closer interaction in 1T MoS_2_.

Taking a step further, Linghu et al. studied the role of defects in both 2H and 1T polytype and found again that defective 1T-MoS_2_ is superior in NO_2_ adsorption [287]. The single S vacancy defects are considered in both phases due to their low formation energy requirement.

**Fig. 7:**
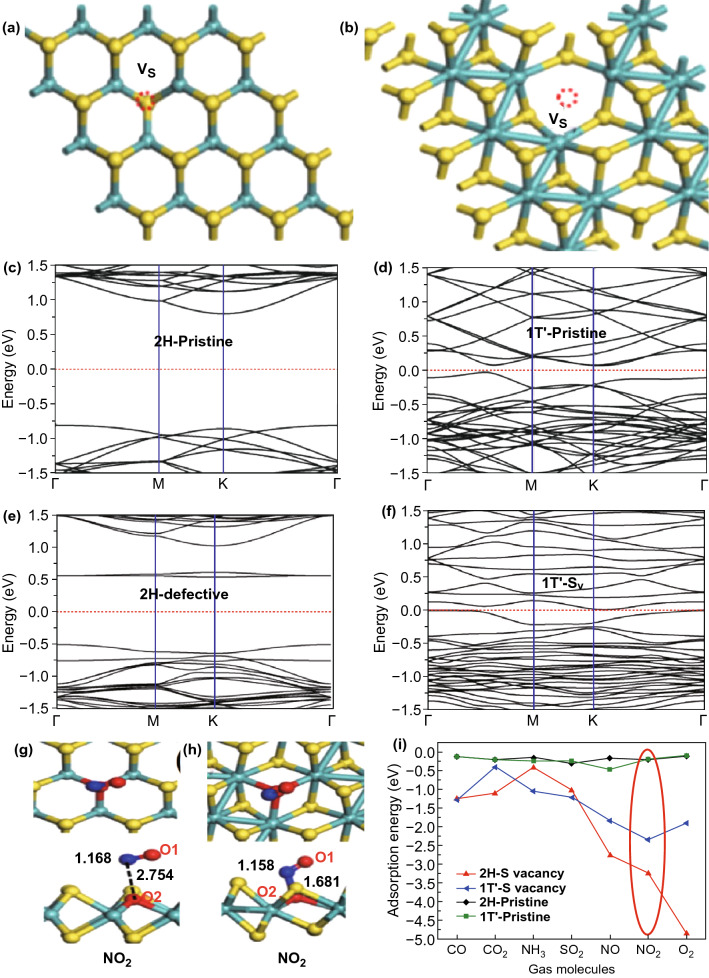
**a** S vacancy in 2H-MoS_2_. **b** S vacancy in 1T-MoS_2_. Band structure of **c** pristine 2H-MoS_2_, **d** pristine 1T-MoS_2_, **e** S vacant 2H-MoS_2_, **f** S vacant 1T-MoS_2_. Adsorption of NO_2_ on **g** defective 2H-MoS_2_, **h** defective 1T-MoS_2_. **i** Variation of adsorption energy for different molecules. Reproduced from Ref. [286]. Copyright (2019) American Chemical Society

Figure 7a, b demonstrates the geometric perspective structure with S vacancy of 2H and 1T MoS_2_. The S vacancies in both 2H and 1T phase affect the electronic structure of the MoS_2_. S vacancies introduced mid gaps states and further reduced the MoS_2_ bandgap. Moreover, the metallic behavior of 1T-MoS_2_ is increased due to these mid gap states. The band structure of 2H and 1T MoS_2_ of pristine and defective MoS_2_ are shown in Fig. 7c–f. When NO_2_ is exposed to these polytypes, it dissociates in NO and O, as shown in Fig. 7g, h. The O atom tri-coordinated with the neighboring three Mo atom and occupied the S vacancy site and NO gets physisorbed on MoS_2_. The variation of adsorption energy with different molecules is shown in Fig. 7i. The red encircled values depict the NO_2_ adsorption energies.

### *Theoretical Adsorption of NO*_*2*_* Gas Molecules on Metal-Functionalized MoS*_*2*_

The absence of dangling bonds makes the pristine monolayer MoS_2_ surface defects free. However, the defects are highly probable and S vacancies are the most favorable defect due to the less energy required for their formation [102, 288–290]. There are various experimental reports in which S defects have been controlled by using the argon and electron irradiation. Filling these mono vacancy sites with substitutional atoms can be a promising way to enhance the chemical, electrical and optical properties of MoS_2_ layers [289, 291]. These vacancy sites have been filled with various metal atoms such as Cr, Nb, V, and N, experimentally and the electronic and chemical activities of MoS_2_ layers changed dramatically [291–293]. Yuan et al. doped graphene with Al, Si, Cr, and Mn and studied the oxygen adsorption on the metal-doped graphene using DFT. The metal doping tuned the adsorption interaction of oxygen with carbon atoms of graphene. The bonding of the metal atom with the carbon atom is a responsible factor for enhance oxygen adsorption on the doped graphene [294]. Lu et al. embedded the graphene with Au and investigated the CO oxidation using DFT [295]. Au embedding reduces the reaction barrier and increases the oxidation rate of the CO on Au embedded graphene. Similarly, the inert 2D materials surface can be changed to a highly active surface for gas interaction due to the bonding of 2D materials with metal atoms.

Therefore, metal doping has a great impact on the electronic and gas-sensing properties such as adsorption energy, charge transfer, the direction of charge transfer and interaction of gas molecules with the MoS_2_ surface. The choice of appropriate metal for a particular gas will strongly modulate the chemical activity, selectivity and sensor response of the MoS_2_ surface.

Fan et al. investigated the effect of transition metals (Fe, Co, Ni, Cu, Ag, Au, Rh, Pd, Pt, and Ir) doping on MoS_2_ flakes for various gas molecules adsorption (CO, NO, O_2_, NO_2_, and NH_3_). The effect of transition metal doping in the absence of the gas molecules has been systematically studied. All the mentioned metals have been doped on the mono-sulfur vacancy site due to the low formation energy of S vacancies in comparison to other vacancies such as Mo vacancy, dia Mo vacancy and antisite vacancies [102, 288–290]. The equilibrium height (M-Mo) is taken from the metal atom and S atom plane. The stability of the metal embedded MoS_2_ in terms of binding energy and charge transfer was tested to have a better grasp. The binding energy ($E_{{\text{b}}}$) between the metal atom and unexposed MoS_2_ is calculated by Eq. (5):

5 $$ E_{{\text{b}}} = E_{{{\text{MoS}}_{2} }} + E_{{{\text{metal}}}} - E_{{{\text{MoS}}_{2} + {\text{metal}}}} $$

The highest binding energy (energy required to bind the metal atom on the S vacant MoS_2_) was found 5.21 eV for Pt metal atoms and the lowest for 1.98 eV for the Ag atoms. The maximum charge 0.36e was transferred from Fe metal to MoS_2_ and the lowest − 0.34e to Pt metal atom from MoS_2_. The negative charge value means transition metals obtain the electrons from the MoS_2_ and vice versa for positive charge value. The binding energy and charge transfer values mentioned above are without NO_2_ exposure. The charge depletion and accumulation between the metals and MoS_2_ are due to the Pauling electronegativity. For the case of NO_2_ adsorption on the metal-doped MoS_2_ sheet, two different modes were obtained after the relaxation of the exposed MoS_2_ system. One mode is with Fe, Co, Cu, Ag, and Au embedded MoS_2_ system via bonding of two O atoms with transition metals forming TM–O–N–O (four membered ring). The other mode is the bonding of NO_2_ with Ni-, Rh-, Pd-, Pt-, and Ir-doped MoS_2_ in which N-atom bonded with the transition metal. The adsorption energies and charge transfer in case of NO_2_ adsorbed on the metal-doped MoS_2_ are tabulated in Table 2. Fan et al. calculated the adsorption energy of gas molecules by Eq. (6):

6 $$ E_{{\text{a}}} = E_{{{\text{free}}\,{\text{molecule}}}} + E_{{{\text{free}}\,{\text{sheet}}}} - E_{{{\text{adsorbed}}\,{\text{sheet}}}} $$

**Table 2:** Summary of the adsorption energy, charge transfer, and method utilized for the calculating the NO_2_ adsorption on the various metal-doped

Metal	Supercell Size (lattice parameter in Å)	Method	$E_{{{\text{ads}}}}$ (eV)	Charge transfer (e)	References
Fe	4 × 4 (3.18) $E_{{\text{a}}} = E_{{{\text{free}}\,{\text{molecule}}}} + E_{{{\text{free }}\,{\text{sheet}}}} - E_{{{\text{adsorbed}}\,{\text{sheet}}}}$	PBE*	1.92	− 0.66	[296]
Co			1.45	− 0.61	
Ni			0.84	− 0.42	
Cu			1.02	− 0.64	
Ag			0.54	0.60	
Au			0.65	− 0.54	
Rh			1.13	− 0.31	
Pd			0.29	− 0.34	
Pt			0.37	− 0.34	
Ir			1.49	− 0.39	
MoS_2_	4 × 4 (3.17) $E_{{\text{a}}} = E_{{{\text{MoS}}_{2} + {\text{molecule}}}} - \left( {E_{{{\text{MoS}}_{2} }} + E_{{{\text{molecule}}}} } \right)$	PBE	− 0.07	− 0.02	[297]
Al			− 3.02	− 0.50	
Si			− 2.58	− 0.52	
P			− 2.134	− 0.48	
V	4 × 4 (3.17) $E_{{\text{a}}} = E_{{{\text{free}}\,{\text{molecule}}}} + E_{{{\text{free }}\,{\text{sheet}}}} - E_{{{\text{adsorbed}}\,{\text{sheet}}}}$	PBE	2.59	− 0.66	[298]
Nb			3.88	− 0.69	
Ta			3.64	− 0.72	
Ag	4 × 4 (3.18) $E_{{\text{a}}} = E_{{{\text{MoS}}_{2} + {\text{molecule}}}} - \left( {E_{{{\text{MoS}}_{2} }} + E_{{{\text{molecule}}}} } \right)$	PBE	− 2.83	− 0.61	[299]
Cu	5 × 5 (3.18) $E_{{\text{a}}} = E_{{{\text{free}}\,{\text{molecule}}}} + E_{{{\text{free }}\,{\text{sheet}}}} - E_{{{\text{adsorbed}}\,{\text{sheet}}}}$	PBE	1.66	0.64	[25]

^*^Perdew–Bruke–Ernzerh (PBE); The negative value means charge transfers from MoS_2_ to gas molecules; the doping site is at S vacancies for all the reports

**Fig. 8:**
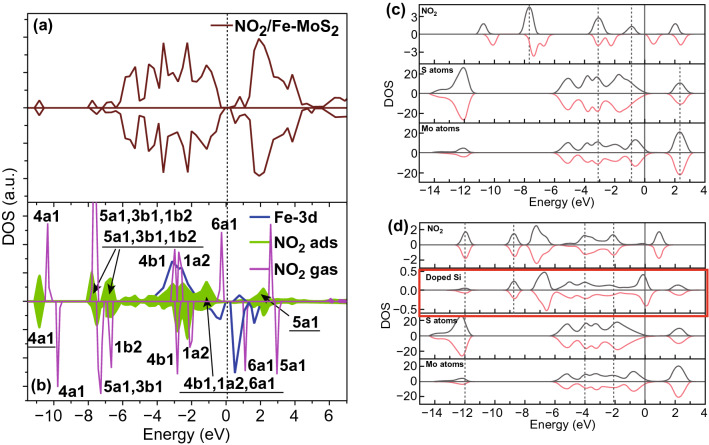
**a, b** Total density of states and density of states for Fe-embedded NO_2_ molecule. Reproduced with permission from Ref. [296]. Copyright (2017) Elsevier; Calculated projected density of states **c** with NO_2_ adsorbed on monolayer MoS_2_, **d** Si-doped MoS_2_. Reproduced with permission from Ref. [297]. Copyright (2016) Elsevier

The Fe metal-embedded MoS_2_ has shown promising NO_2_ adsorption properties with charge transfer value − 0.66e and adsorption energies of 210 meV. The negative values indicate that charge transferred from metal embedded MoS_2_ to NO_2_ than pristine MoS_2_. These extra electrons are obtained from the embedded transition metals, which reflect the importance of the transition metals. The electronic structure with NO_2_ and metal embedded MoS_2_ was studied deeply. The higher interaction of NO_2_ is due to the mixing of Fe 3d states and 6a1, 1a2, and 4b1 orbitals of NO_2_ over a wide range of energy, as shown in the Fig. 8a, b. These mixing or hybridization resulted in enhanced NO_2_ interaction with charge transfer of − 0.66e. A similar behavior is observed with other metal-doped MoS_2_.

Luo et al. doped Al, Si, and P metal atoms at the S vacancy site [297]. These metals were chosen because of their exactness and closeness of covalent radii to the radius of the S atom. The NO_2_ and NH_3_ adsorption were studied at five adsorption sites on MoS_2_. The five adsorption sites are $$T_{x}$$ (gas molecule on top of doped metal), $$H_{x}$$ (gas molecule on top of hexagon near to doped metal), $$T_{{\text{S}}}$$ (gas molecule on top of S atom near to doped metal), $$T_{{{\text{Mo}}}}$$ (gas molecule on top Mo atom near to doped metal). Among all five sites, the most stable site for NO_2_ adsorption was $$ H_{x}$$ after a complete structure relaxation. The doping of Al, Si, and P generates impurities in the Mo 4d state which create strong hybridization coupling between the Al-3p, Si-3p, and P-3p. Therefore a strong charge is transferred between the atoms and monolayer MoS_2_. Si-doped MoS_2_ was found most suitable for NO_2_ adsorption due to the highest charge transfer between them. PDOS calculation was performed to investigate the NO_2_ adsorption on undoped MoS_2_ and doped MoS_2_, and shown in Fig. 8c, d. In the case of undoped MoS_2_, the NO_2_ peaks were situated at − 7.7 and − 3.09 eV while the PDOS peak of bare MoS_2_ was situated at 2.33, − 12.04 and between − 1.5 and − 5 eV. Hence the weak interaction occurs between NO_2_ and MoS_2_. However, when Al was doped in MoS_2_, there is more orbital coupling at − 1.35 and − 3.31 eV not only with Al orbitals but also with S and Mo orbitals. Hence, the interaction and charge transfer increased with Al doping. NO_2_ molecules partially obtained electrons from the doped Al. With Si atom, the hybridization of orbitals is further increased and a higher number of electrons, i.e., 0.52e transfer to MoS_2_. Similar behavior was observed with the P atom.

**Fig. 9:**
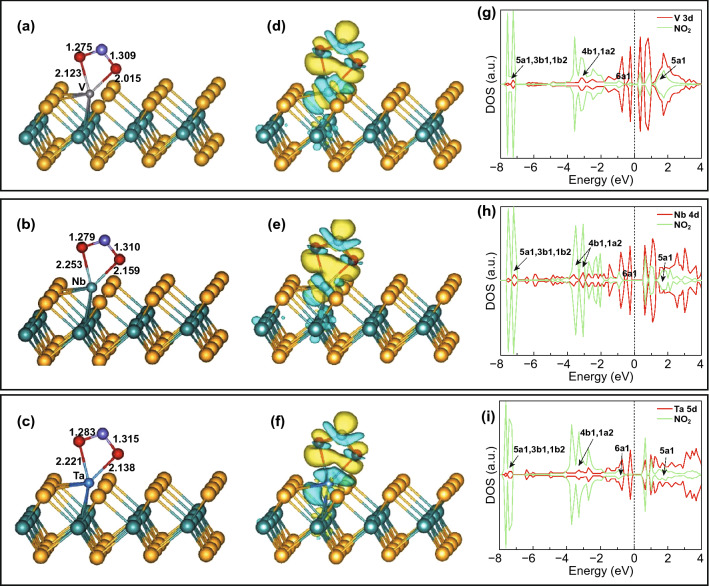
**a, d, g** NO_2_ molecule adsorbed on V metal: optimized geometry after NO_2_ adsorption (**a**)**,** charge density difference (**d**), spin-polarized density of state with V 3d and NO_2_ (**g**). **b, e, h** NO_2_ molecule adsorbed on Nb metal: optimized geometry after NO_2_ adsorption (**b**), charge density difference (**e**), spin-polarized density of state with Nb 4d and NO_2_ (**h**). **c, f, i** NO_2_ molecule adsorbed on Ta metal: optimized geometry after NO_2_ adsorption (**c**), charge density difference (**f**), spin-polarized density of state with Ta 5d and NO_2_ (**i**). Reproduced with permission from Ref. [298]. Copyright (2017) Elsevier

Zhu et al. studied the doping of V, Tb, and Ta on the S vacancy site [298]. It is important to note that the size of these metal atoms is large in comparison to the S atom. These atoms are thus situated outside the S plane. Among all, the high binding energy suggested that Ta atoms bound firmly with MoS_2_. The NO_2_ gas molecules prefer to make bond on metal atoms. The two oxygen atoms form bond with the metal atom and N atom, and form a four-membered ring like structure M–O–N–O, shown in Fig. 9a–c. The calculated adsorption energies were 2.59, 3.88, and 3.64 eV for V, Nb, and Ta atoms, respectively. The Bader charge analysis revealed that charge transferred from MoS_2_ to NO_2_ and with V, Nb, and Ta atoms metals doping. NO_2_ has shown strong oxidizing behavior. The charge density differences are shown in Fig. 9d–f. The NO_2_ adsorption with monolayer MoS_2_ were further calculated with NO_2_ exposure.

However, the charge transfer and adsorption energies are comparatively smaller than metal-doped V, Nb, and Ta. Moreover, NO_2_ as a paramagnetic molecule is critically affected by the bond length [300]. The bond length was 1.21 Å in the case of pristine MoS_2_ while NO_2_ bond length was elongated from 0.07 to − 0.11 Å with metal-doped MoS_2_. Thus, the NO_2_ activation on metal-doped MoS_2_ is enhanced. Further electronic properties of MoS_2_ after NO_2_ doping was analyzed in terms of DOS, shown in Fig. 9g–i. The metal orbitals and NO_2_ orbitals have a strong hybridization between their orbitals. The d orbitals of metals especially for Nb atoms get mixed with NO_2_ orbitals over a wide range of energy. Hence, doping of MoS_2_ with V, Nb, and Ta improves the electronic and chemical performance of the NO_2_ molecule. The supercell size, lattice parameter, occupied method, adsorption energy, and charge transfer are summarized in Table 2.

## Experimental Investigations of NO_2_ Adsorption on MoS_2_

In this section, we discuss various experimental approaches employed to develop the NO_2_ sensors. This section has been divided into five sub-section in which we summarize the various experimental approaches adopted in terms of bare MoS_2_, morphology-driven MoS_2_, metal-doped MoS_2_, vacancy-driven MoS_2_, and finally light-assisted MoS_2_-based NO_2_ sensors.

### Bare MoS_2_ NO_2_ Sensor

**Fig. 10:**
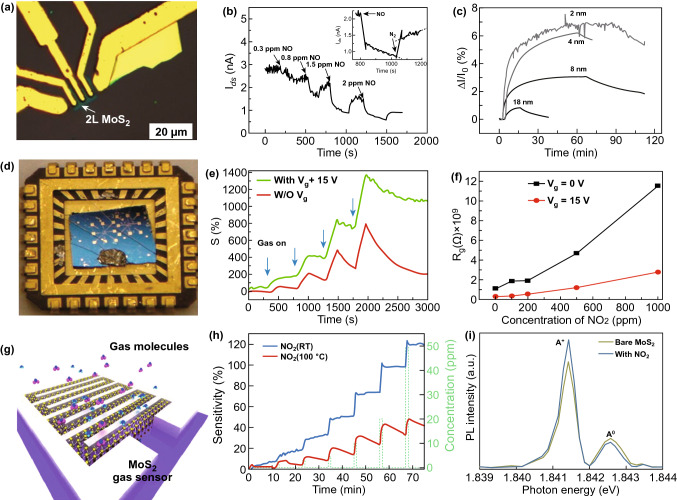
**a** Optical image of bilayer MoS_2_-based FET NO sensor. **b** MoS_2_ FET response to different concentrations NO. The inset showed the typical response and recovery of the MoS_2_ FET device. Reproduced with permission from Ref. [34]. Copyright (2012) Wiley-VCH. **c** MoS_2_ TFT NO_2_ sensor with different thickness MoS_2_ flakes. Reproduced with permission from Ref. [42]. Copyright (2012) Wiley-VCH; **d** optical image of MoS_2_ device mounted on a chip. **e** NO_2_ response for the bi and five-layer MoS_2_ devices at different gate voltages. **f** Theoretically calculated resistance variation with different gate voltages. Reproduced with permission from Ref. [17]. Copyright (2013) American Chemical Society. **g** Device schematic of atomic layer MoS_2_-based sensing device. **h** Response of NO_2_ at RT and at moderate temperature of 100 °C. **i** Change in the low energy PL peak due to NO_2_ adsorption. Reproduced with permission from Ref. [32]. Copyright (2015) Springer Nature

Here, we addressed several efforts and experimental reports where NO_2_ sensors were fabricated with single and multilayered MoS_2_ flakes. The reports include the impact of NO_2_ adsorption on the single and multilayer MoS_2_ and as well as the on the SBH. Li et al. developed the first NO_x_ gas sensor using an n-type MoS_2_ flakes-based FET device [34]. The schematic of fabricated device is shown in Fig. 10a. The monolayer (1L) to quadrilayer (4L) MoS_2_ flakes were synthesized by the mechanical exfoliation technique and had the detection limit of 0.8 ppm. The thickness of the MoS_2_ layers was confirmed by the atomic force microscopy (AFM) technique. The current versus voltage characteristics measurements of the device with varied layers were performed. The single layer device showed unstable behavior while bi- to quadrilayer film-based devices demonstrated better sensing performance. The NO gas exposure to bilayer MoS_2_ film showed a decrease in the current, which confirm the p-type doping due to the electron acceptor nature of the NO gas [57, 301]. Figure 10b displays the gas-sensing performance of the MoS_2_ device with different NO concentrations. The adsorption and desorption rate of NO was a two-step process: fast rate and slower rate. The fast reduction in current confirmed the presence of a large number of NO adsorption sites and slow reduction confirmed saturation of MoS_2_ film in NO exposure. Another significant aspect of the different thickness of MoS_2_ film was the quick response to NO exposure. The single layer MoS_2_ film showed a 50% response within 5 s while multilayer MoS_2_ showed a 50% response in 30 s. However, the disadvantage with single-layer MoS_2_ film was its instability.

He et al. developed a flexible MoS_2_ thin film transistor (TFT) arrays for the NO_2_ sensing [42]. The single layer MoS_2_ film suspension was drop cast over patterned rGO electrodes covered with Ag pads. The Ag pads had only been used to improve the robustness of the rGO electrodes. The MoS_2_ area and thickness for NO_2_ sensing were 1.5 mm^2^ and 2, 4, 8, and 18 nm, respectively. It is worth to note that the deposited MoS_2_ film showed p-type behavior attributing to the structural changes caused by the lithium intercalation process. The structural changes in the MoS_2_ lead to a change in the band structures. During distortion from the octahedral system to zigzag chain, the system was filled up to $d^{2 + n}$ states. Hence, residual negative charges semi filled the bands and contributed to p-type conductivity [302]. The NO_2_ gas exposed to the various thickness of MoS_2_ film and the highest change in the sensor response was occurred for the thinnest MoS_2_ film. The NO_2_ exposure increased the conductance of the film due to its electron acceptor nature. The high NO_2_ detection ability of thin MoS_2_ film was attributed to the increased surface area available in 2 nm film. The sensor response of different thickness of MoS_2_ film is shown in Fig. 10c. Late et al. studied the NO_2_ sensing behavior of single and multiple layer MoS_2_ film synthesized by the mechanical exfoliation method [17]. A detailed gas-sensing performance with and without applying the bias voltage was presented. A detailed AFM, Raman, and TEM characterization were performed to understand the thickness, expansion, crystallographic orientation, and structure of MoS_2_. The device schematic with Ti/Au contact is shown in Fig. 10d. The *I*–*V* characteristic of single layer MoS_2_ device was unstable while multilayer MoS_2_ showed stable *I*–*V* characteristics. Few layers (single and five layers) MoS_2_ device demonstrated good behavior. The three and four-layer MoS_2_ flakes device showed identical behavior to two layer and five-layer devices. The NO_2_ sensing for five-layer MoS_2_ device is shown in Fig. 10e. However, this higher performance was due to the redox potential that greatly influences the sensing behavior of MoS_2_ flakes. Once again, the NO_2_ interaction with MoS_2_ revealed that the NO_2_ has an electron acceptor nature. The influence of the external electric field in terms of bias voltage on the NO_2_ sensing was further studied. When a positive back gate biasing voltage + 15 V was applied to two and five layers of MoS_2_ flakes, the sensor response was improved in comparison to zero bias voltage. A larger number of electrons were collected at the MoS_2_ and SiO_2_ interface under positive back gate voltage. Therefore, NO_2_ has a higher number of electrons to detach from the MoS_2_. With positive gate biasing voltage, the NO_2_ sensor response was thus increased. In addition, Ti/Au electrode played a vital role under positive gate voltage. Under positive gate voltage, electrons get accumulated in MoS_2_ film and the barrier between the electrode and MoS_2_ film is reduced. Thus, the charge transfer in MoS_2_ film facilitated further. The device resistance in the presence of NO_2_ gas is shown in Fig. 10f at different biasing voltages.

Cho et al. synthesized the atomic layered MoS_2_ flakes by the CVD technique and performed the NO_2_ gas sensing [32]. The resistance of the n-type MoS_2_ film increased due to the electron-accepting nature of NO_2_. The interdigitated electrodes of Ag metal were fabricated on the MoS_2_ film. The NO_2_ sensing performance was studied at RT and at a moderate temperature of 100 °C.

The device schematic and NO_2_ sensor response versus time profile at each temperature are shown in Fig. 10g, h. It can be seen clearly that the RT sensor response was quite high in comparison to 100 °C, while the sensor showed rapid recovery at 100 °C and no recovery was obtained at RT. The NO_2_ gas strongly adsorbed on MoS_2_ and hence at RT the desorption rate is quite low. However, thermal energy greatly impacts the adsorption of NO_2_ at a higher temperature. The thermal energy accelerates the NO_2_ desorption rate than the adsorption rate. As a result, the NO_2_ gas interaction decreases at a higher temperature at the sensor response cost. The NO_2_ sensing mechanism based on the charge transfer process, confirmed by the change in the peaks of PL spectra is shown by Fig. 10i, as we discussed in Fig. 5b of Sect. 3.

These all layer-dependent studies show that the single layer MoS_2_-based gas sensors suffered from unstable current, but they have a quick response with NO_2_ exposure. The few layer MoS_2_ flakes-based gas sensors show a good response with the stable current. Moreover, the MoS_2_ FET gas sensors are very sensitive to the applied bias voltage. However, the MoS_2_ gas sensors have an incomplete recovery at RT. So, operating sensors at a higher temperature may be a good option to achieve full recovery but it will reduce the sensor response. The summary of the results for bare MoS_2_-based NO_2_ gas sensors by various groups are tabulated in Table 3.

**Table 3:** Summary of the reported NO_2_ sensor based on the MoS_2_

Sensing film	Type	Method	Def.	Electrodes	Device	Conc. (ppm)	Tem. (°C)	S (%)	Res time	Rec time	References
1L MoS_2_	n	M.E.	$\left( {\frac{{\left( {I_{\text{gas}} - I_{\text{air}} } \right)}}{{I_{\text{air}} }}} \right)$	Ti/Au	FET	2	RT	80	–	–	[34]
MoS_2_ sheets	p	E.L.	$\left( {\frac{{\left( {R_{\text{gas}} - R_{\text{air}} } \right)}}{{R_{\text{air}} }}} \right)$	rGO	TFT	1.2	RT	7	–	–	[42]
5L MoS_2_	n	M.E.	$\left( {\frac{{\left( {R_{\text{gas}} - R_{\text{air}} } \right)}}{{R_{\text{air}} }}} \right)$	Ti/Au	FET	1000	RT	1372	300	600	[17]
1L MoS_2_	n	CVD	$\left( {\frac{{\left( {G_{\text{gas}} - G_{\text{air}} } \right)}}{{G_{\text{air}} }}} \right)$	Ti/Au	FET	.02	RT	> 20	300	–	[36]
3L MoS_2_	p	CVD	$\left( {\frac{{\left( {I_{\text{gas}} - I_{\text{air}} } \right)}}{{I_{\text{air}} }}} \right)$	Al	Resistor	10	RT	80	–	–	[303]
2L MoS_2_	p	CVD	$\left( {\frac{{\left( {I_{\text{gas}} - I_{\text{air}} } \right)}}{{I_{\text{air}} }}} \right)$	Ag	Resistor	10	RT	98	–	–	[303]
4L MoS_2_	p	CVD	$\left( {\frac{{\left( {I_{\text{gas}} - I_{\text{air}} } \right)}}{{I_{\text{air}} }}} \right)$	Au	Resistor	10	RT	60	–	–	[303]
Atomic layered MoS_2_	n	CVD	$\left( {\frac{{\left( {R_{\text{gas}} - R_{\text{air}} } \right)}}{{R_{\text{air}} }}} \right)$	Ag	Resistor	50	RT	~ 120	–	–	[32]
Atomic layered MoS_2_	n	CVD	$\left( {\frac{{\left( {R_{\text{gas}} - R_{\text{air}} } \right)}}{{R_{\text{air}} }}} \right)$	Ag	Resistor	50	100	> 40	–	–	[32]
Few layer MoS_2_	p	L.E.	$\frac{{R_{\text{gas}} }}{{R_{\text{air}} }}$	Pt	Resistor	1	200	1.15	660	720	[31]
Few layer MoS_2_	n	L.E.	$\frac{{R_{\text{gas}} }}{{R_{\text{air}} }}$	Pt	Resistor	1	200	5.80	2460	2340	[31]
Vertical MoS_2_	p	CVD	$\left( {\frac{{\left( {R_{\text{gas}} - R_{\text{air}} } \right)}}{{R_{\text{air}} }}} \right)$	Ti/Au	Resistor	100	RT	> 10	–	–	[35]
Vertical MoS_2_	n	CVD	$\left( {\frac{{\left( {R_{\text{gas}} - R_{\text{air}} } \right)}}{{R_{\text{air}} }}} \right)$	Au/Cr	Resistor	50	RT	48.32	98	–	[304]
Vertical MoS_2_	n	CVD	$\left( {\frac{{\left( {R_{\text{gas}} - R_{\text{air}} } \right)}}{{R_{\text{air}} }}} \right)$	Au/Cr	Resistor	50	RT	24.26	34	132	[304]
2L MoS_2_	p	CVD	$\left( {\frac{{\left( {R_{\text{gas}} - R_{\text{air}} } \right)}}{{R_{\text{air}} }}} \right)$	Au	Resistor	1	RT	2.6	678	318	[305]
Mixed MoS_2_	p	CVD	$\left( {\frac{{\left( {R_{\text{gas}} - R_{\text{air}} } \right)}}{{R_{\text{air}} }}} \right)$	Au/Cr	Resistor	10	RT	10.36	8.51	–	[120]
Mixed MoS_2_	p	CVD	$\left( {\frac{{\left( {R_{\text{gas}} - R_{\text{air}} } \right)}}{{R_{\text{air}} }}} \right)$	Au/Cr	Resistor	10	125	7.79	4.44	19.6	[120]
MoS_2_ NWs	n	CVD	$\left( {\frac{{\left( {R_{\text{air}} - R_{\text{gas}} } \right)}}{{R_{\text{air}} }}} \right)$	Au	Resistor	5	60	18.1	16	172	[214]
MoS_2_ nanoflower	p	Hyd.	$\frac{{R_{\text{gas}} }}{{R_{\text{air}} }}$	Au	Resistor	50	150	78	–	–	[33]
MoS_2_ nanosphere/CTAB	n	Hyd.	$\left( {\frac{{\left( {R_{\text{air}} - R_{\text{gas}} } \right)}}{{R_{\text{air}} }}} \right)$	Ag/Pd	Resistor	50	150	60	15	12	[306]
MoS_2_ hollow microsphere	p	Hyd.	$\left( {\frac{{\left( {R_{\text{air}} - R_{\text{gas}} } \right)}}{{R_{\text{air}} }}} \right)$	Au/Cr	Resistor	100	150	40.3	79	225	[307]
MoS_2_ Nanoflowers	p	Hyd.	$\left( {\frac{{\left( {R_{\text{air}} - R_{\text{gas}} } \right)}}{{R_{\text{air}} }}} \right)$	Pt	Resistor	5	RT	67.4	125	485	[308]
MoS_2_ Nanoflowers	p	Hyd.	$\left( {\frac{{\left( {R_{\text{air}} - R_{\text{gas}} } \right)}}{{R_{\text{air}} }}} \right)$	Pt	Resistor	5	150	22	95	320	[308]
MoS_2_ Aerogel	p	T.D.	$\left( {\frac{{\left( {R_{\text{gas}} - R_{\text{air}} } \right)}}{{R_{\text{air}} }}} \right)$	Pt/Ti	Resistor	0.5	200	11	33	107	[24]
MoS_2_ Nanosheets	p	M.E.	$\frac{{R_{\text{gas}} }}{{R_{\text{air}} }}$	Ag/Pd	Resistor	100	RT	29	42	2	[309]
MoS_2_-W	n	Hyd.	$\left( {\frac{{\left( {R_{\text{air}} - R_{\text{gas}} } \right)}}{{R_{\text{air}} }}} \right)$	Ag/Pd	Resistor	20	RT	171	41	39	[121]
MoS_2_-Au	p	S.P.	$\left( {\frac{{\left( {R_{\text{gas}} - R_{\text{air}} } \right)}}{{R_{\text{air}} }}} \right)$	Au	Resistor	2.5	RT	30	240	840	[121]
rGO/Sv-MoS_2_	p	CVD	$\left( {\frac{{\left( {R_{\text{gas}} - R_{\text{air}} } \right)}}{{R_{\text{air}} }}} \right)$	Au	Resistor	50	50	72	56	328	[310]
MoS_2_	n	CVD	$\left( {\frac{{\left( {R_{\text{gas}} - R_{\text{air}} } \right)}}{{R_{\text{air}} }}} \right)$	Au	Resistor	50	50	27	142	–	[310]
Multi MoS_2_	n	CVD	$\left( {\frac{{\left( {R_{\text{gas}} - R_{\text{air}} } \right)}}{{R_{\text{air}} }}} \right)$	Au/Cr	Resistor	100	RT	27.92	249	–	[119]
Multi MoS_2_	n	CVD	$\left( {\frac{{\left( {R_{\text{gas}} - R_{\text{air}} } \right)}}{{R_{\text{air}} }}} \right)$	Au/Cr	Resistor	100	100	21.56	71	310	[119]
MoS_2_-Au NPs	p	Redox	$\left( {\frac{{\left( {R_{\text{gas}} - R_{\text{air}} } \right)}}{{R_{\text{air}} }}} \right)$	Au	Resistor	2.5	RT	30	240	840	[121]

Mechanical exfoliation (M.E.); liquid exfoliation (L.E.); electrochemical lithiation (E.L.); hydrothermal (Hyd.); thermal decomposition (T.D.); solution processed (S.P.)

**Fig. 11:**
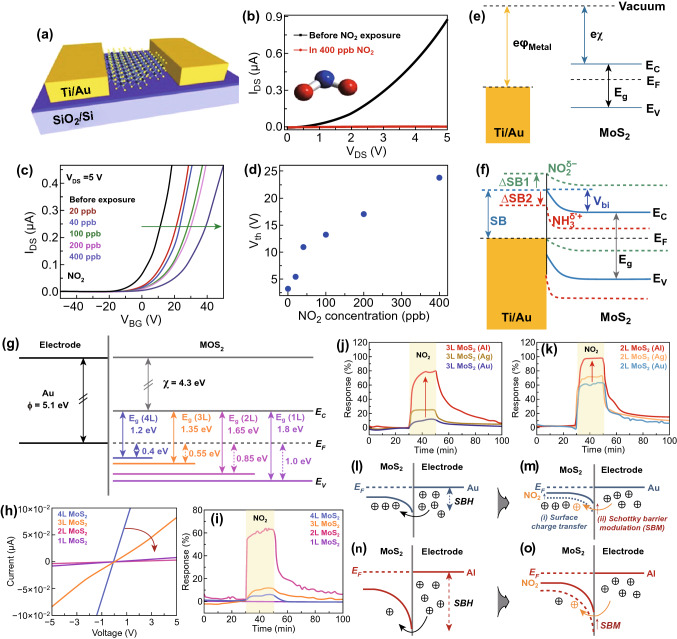
**a** Schematic of the monolayer MoS_2_ device. **b** Change in current after 400 ppb NO_2_ exposure. **c** *I*–*V* characteristics after the NO_2_ exposure of different concentration. **d** A monotonic shift in threshold voltage towards the positive side of applied voltage. **e, f** Energy band alignment before and after NO_2_ exposure. The blue solid lines are corresponding to the band alignment of MoS_2_ and Ti/Au contact in the absence of NO_2_ while the green dotted lines are corresponding to NO_2_ exposure. Reproduced with permission from Ref. [36]. Copyright (2014) American Chemical Society. **g** Band alignment of MoS_2_ with Au metal contact. **h** Current versus voltage characteristics with gold contact. **i** NO_2_ response with MOS_2_-Au device. Sensor response for NO_2_ with different metal contacts Al, Ag, and Au electrodes: **j** 3L MoS_2_ film, **k** 2L MoS_2_ film. Proposed band alignment of MoS_2_-Au device: **l** before, **m** after NO_2_ exposure. Proposed band alignment of MoS_2_-Al device: **n** before, **o** after NO_2_ exposure. Reproduced with permission from Ref. [303]. Copyright (2019) American Chemical Society. (Color figure online)

Liu et al. studied the NO_2_ sensing efficiency of monolayer MoS_2_ flakes grown by CVD [36, 303]. The effect of gas molecules adsorption on the Schottky barrier height (SBH) between the MoS_2_ and metal electrodes was studied. The sensing device area was 1 µm^2^ and film showed the 3 cm^2^ V^−1^ s^−1^ mobility with Ti/Au electrodes, shown in Fig. 11a. The Ti was used for improving the electrode adhesion with MoS_2_ film. The device showed highly rectifying behavior with a positive and negative drain to source voltage (*V*_DS_) with 400 ppb NO_2_ exposure, as shown in Fig. 11b. The device showed an excellent sensor response of 174% with back gate voltage 30 V. The response time was 300 to 540 s with the full recovery in 12 h. To confirm the NO_2_ gas-sensing mechanism via the charge transfer process, the back gate voltage was fixed at 5 V and gas concentration was varied from 20 to 400 ppb. The threshold voltage for the NO_2_ sensing received a monotonic shift in the positive *V*_DS_ direction. The resistance modulation in the device due to gas exposure is the sum of channel resistance $(R_{{{\text{channel}}}} )$ and $R_{{{\text{contact}}}}$ determined by Eq. (7):

7 $$ R = \left( {R_{{{\text{channel}}}} \propto {\raise0.7ex\hbox{$1$} \!\mathord{\left/ {\vphantom {1 n}}\right.\kern-\nulldelimiterspace} \!\lower0.7ex\hbox{$n$}}} \right) + \left( {R_{{{\text{contact}}}} \propto \frac{1}{n}e^{{\frac{{\varphi_{{{\text{SB}}}} }}{kT}}} } \right) $$

where *n* is the electron concentration, $\varphi_{{{\text{SB}}}}$ is the Schottky barrier height between the MoS_2_ and metal electrodes. The SBH is greatly influenced by the above equation when the electron concentration in the device is changed. Figure 11c, d indicates the transfer characteristics at a fixed back gate voltage of 5 V. An increment in the threshold voltage with higher gas concentration is observed. NO_2_ is strong oxidizing gas and has an electron acceptor nature. More number of electrons withdraw from MoS_2_ film as the gas concentration is increased. Thus, a monotonic shift in the positive *V*_DS_ direction was observed. Considering the effect of the NO_2_ adsorption on SBH, the proposed band alignment before and after exposure to NO_2_ is shown in Fig. 11e, f. NO_2_ gas captured the electrons from the conduction band and the electron concentration in MoS_2_ film was decreased. The decrease in electron concentration shifts the Fermi level towards the valence band which increases the SBH. Hence, the conductance is decreased.

Kim et al. recently fabricated the MoS_2_ gas sensor with different metal contacts of different work functions [303]. The sensor response of the MoS_2_ sensor was different with different metals. First, the effect of the layer thickness from single to four layers with Au electrodes was studied. The *I*–*V* characteristic is shown in Fig. 11h revealed a linear behavior and a decrease in resistance with an increase in layer observed. The work-function was increased with the number of layers, shown in Fig. 11g. Hence, for a higher number of layers, the SBH is decreased and according to Eq. 11, the resistance is also decreased. Further, NO_2_ exposure on different thickness layer devices is also displayed in Fig. 11i. The device showed p-type behavior to NO_2_ exposure. Further, the bilayer MoS_2_ device showed the highest sensor response for 10 ppm NO_2_ concentration up to 60%. Finally, for bilayer and trilayer MoS_2_, the Au ($\emptyset_{M} = 5.1\,{\text{eV}}$), Al ($\emptyset_{M} =$ 4.06 eV), and Ag ($\emptyset_{M} = 4.26\,{\text{eV}}$) electrodes were used. Among all, aluminum electrode-based sensing device showed promising sensor response, 80% for bilayer and 98% for trilayer MoS_2_-based device. Conclusively, the device with lower work function metal electrodes showed better performance. The band alignment between the aluminum (lower work function metal) and MoS_2_ is responsible for high performance as shown in Fig. 11l–o. The SBH is higher for Al electrodes than the Au electrodes. Under positive biasing, a higher number of holes are transferred from Au electrode due to the low SBH. When NO_2_ gas is exposed, the SBH decreases with a decrease in electron depletion due to the p-type nature. Relatively, the ratio of charge transferred in Al/MoS_2_ device is higher than the Au/MoS_2_. Hence, better performance is observed.

These reports confirmed that sensing response is critically affected by the SBH. In chemiresistance gas sensors, the SBH is modulated with gas molecules adsorption due to charge transfer between the molecules and sensing film. Thus, Schottky contacted devices are a good candidate for fabricating gas sensors. Hence, the choice of metal contacts played an important role in gas sensing.

### Morphology-Driven NO_2_ Sensors

In the 2D materials, especially in MoS_2_, morphology plays a vital role in determining the optical, electrical, and catalytic properties. The NO_2_ molecule adsorption in MoS_2_ is position-dependent and there are specific NO_2_ favourable sites for molecules adsorption in MoS_2_. These favourable NO_2_ adsorption sites can be controlled by synthesizing various MoS_2_ film surface morphology. In this section, we will discuss various reports where morphology-dependent NO_2_ sensors based on MoS_2_ developed.

**Fig. 12:**
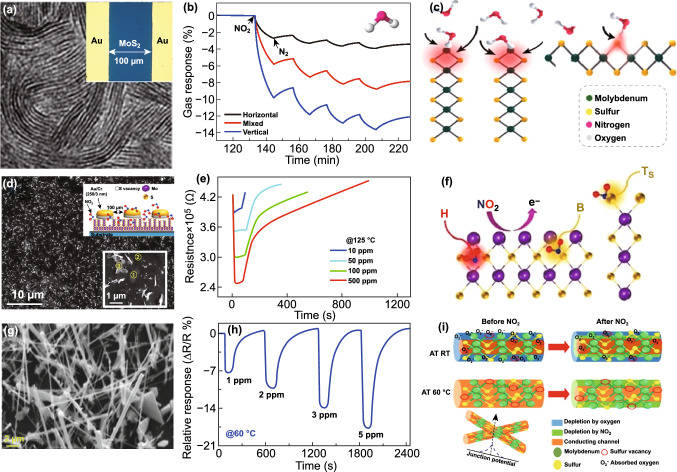
**a** TEM image of the vertically grown MoS_2_. **b** Response of various morphology MoS_2_ flakes with NO_2_ gas. **c** DFT calculated NO_2_ adsorption profile on the edges and basal plane MoS_2_. Edges have high adsorption of MoS_2_ flakes. Reproduced with permission from Ref. [35]. Copyright (2015) American Chemical Society. **d** FESEM image of mixed MoS_2_ flakes and inset showed the high-resolution image of MoS_2_ flakes and the device schematic. **e** The response of mixed MoS_2_ flakes with NO_2_ gas at 125 °C. **f** Schematic of favorable adsorption sites on the MoS_2_ flakes. Reproduced with permission from Ref. [120]. Copyright (2018) American Chemical Society. **g** FESEM image of the grown MoS_2_ NWs. **h** Response of MoS_2_ NWs with NO_2_ exposure. **i** Proposed a mechanism of NO_2_ adsorption on the MoS_2_ NWs. Reproduced with permission from Ref. [214]. Copyright (2018) AIP Publishing

Cho et al. studied the role of MoS_2_ edges in NO_2_ gas molecules adsorption [35]. The orientation of the MoS_2_ film greatly affects the adsorption of NO_2_ molecules. Authors varied the orientation of the MoS_2_ film from horizontal to vertical align by depositing different thickness Mo films. The surface topography is shown in Fig. 12a. The inset of the Fig. 12a showed the schematic of the sensing device with an active area of 100µm^2^. The NO_2_ gas molecule adsorption enhanced up to fivefold in vertical aligned MoS_2_ flakes compared to the horizontal MoS_2_ film, as shown in Fig. 12b. The Mo film was deposited through an electron beam evaporator and was sulfurized in the CVD. The orientation of the MoS_2_ film was determined through the FESEM, XRD, TEM, and Raman spectra. The MoS_2_ films (horizontal, mixed, and vertical MoS_2_) showed a p-type nature. The p-type behavior was verified through the positive increase in the resistance due to the exposure of oxidizing NO_2_ gas. Interestingly, vertical aligned MoS_2_ flakes faced the highest change in the sensor response to the NO_2_ gas, which means that the morphology of MoS_2_ flakes crucially regulates the gas-sensing behavior. The reason is the presence of numerous active sites at the edges.

As we discussed in Sect. 3, the horizontal (basal plane) and vertical aligned MoS_2_ flakes have different adsorption sites (H site, T_S_ site, and T_M_ site) for NO_2_ molecules with different adsorption energy and charge transfer. Moreover, the edges of vertical aligned MoS_2_ flakes have high catalytic properties in comparison with the basal plane, which enhanced the NO_2_ reactivity of the edges. The vertical aligned MoS_2_ flakes thus displayed the great potential to communicate with NO_2_. The adsorption of NO_2_ on the basal plane MoS_2_ and at the edges is shown in Fig. 12c.

Kumar et al. synthesized the horizontally aligned MoS_2_ (HA-MoS_2_) and vertically aligned (VA-MoS_2_) by the CVD method [304]. The NO_2_ sensing behavior for each structure was determined in the operating temperature range from RT to 150 °C. The VA-MoS_2_ flakes showed better NO_2_ sensing performance in all temperature range. Moreover, the VA MoS_2_ film quickly detected 1 ppm NO_2_ concentration. However, the sensor response for 1 ppm NO_2_ concentration with HA MoS_2_ flakes could not be achieved. These results revealed the high NO_2_ detection ability of VA MoS_2_ flakes even for the low concentration also. Another fascinating aspect of VA-MoS_2_ is the sensor recovery after NO_2_ exposure which didn't occur with the HA-MoS_2_ flakes. The recovery of the VA and HA-MoS_2_ flakes was substantially improved by operating devices at high temperatures but again at the expense of sensor response. Notably, the NO_2_ selectivity of the VA-MoS_2_ device was also high. The high sensor response of VA-MoS_2_ flakes is due to the high adsorption sites and the higher number of charge transfer at the edges of the VA-MoS_2_ flakes in comparison with HA-MoS_2_ flakes.

Agrawal et al. synthesized a combination of vertical aligned MoS_2_ flakes and in-plane MoS_2_ flakes (mixed MoS_2_ flakes) by a modified CVD technique. The surface morphology is shown in Fig. 12d. The black region is the in-plane MoS_2_ flakes while the white region is the vertical MoS_2_ flakes. The fabricated sensing system suggested the existence of the p-type nature of MoS_2_ film. The resistance of the device was decreased with the exposure of oxidizing NO_2_ gas which means there is a decrease in the electron concentration and simultaneously an increase in the hole concentrations. The transient response curve with NO_2_ exposure at 125 °C is shown in Fig. 12e. The NO_2_ detection at RT was also studied. However, full recovery could not be achieved. The sensing mechanism of NO_2_ interaction is based on the favorable adsorption sites available on the MoS_2_ flakes, shown in Fig. 12f. MoS_2_ has four adsorption sites as we discussed in Sect. 3, H site, B site, T_M_, and T_S_ site. Yue et al. theoretically showed that the H site, T_M_ site, and B site are the most favorable sites for the NO_2_ adsorption. The maximum combination of these sites was synthesized to obtain the selective, highly responsive and recoverable NO_2_ sensor.

Kumar et al. synthesized the MoS_2_ nanowire through the controlled turbulent vapor flow, shown in Fig. 12g. The NO_2_ sensing behavior of the n-type MoS_2_ NWs was investigated at the RT, 60 °C, and 120 °C for NO_2_ concentrations of 1, 2, 3, and 5 ppm, shown in Fig. 12h. The MoS_2_ NWs showed a high sensor response with an incomplete recovery due to the strong bonding of NO_2_ molecules with NWs. A moderate temperature of 60 °C helped the MoS_2_ NWs to obtain a recovery. The MoS_2_ NWs showed good response time (16 s) and recovery time (172 s) for the 5 ppm NO_2_ concentration with sensor response 18% at 60 °C. The NO_2_ sensing mechanism proposed in MoS_2_ NWs is based on the physisorption and chemisorption of gas molecules, as shown in Fig. 12i. The humidity and environmental oxygen get adsorbed on the surface of the NWs and reduced the detection of the NO_2_ gas molecule at the RT. However, at 60 °C, the humidity and adsorbed oxygen were removed and generated new active sites for the NO_2_ adsorption. Hence, NO_2_ detection was high at a moderate temperature. Moreover, the high temperature generates thermal energy which also helps in the recovery.

**Fig. 13:**
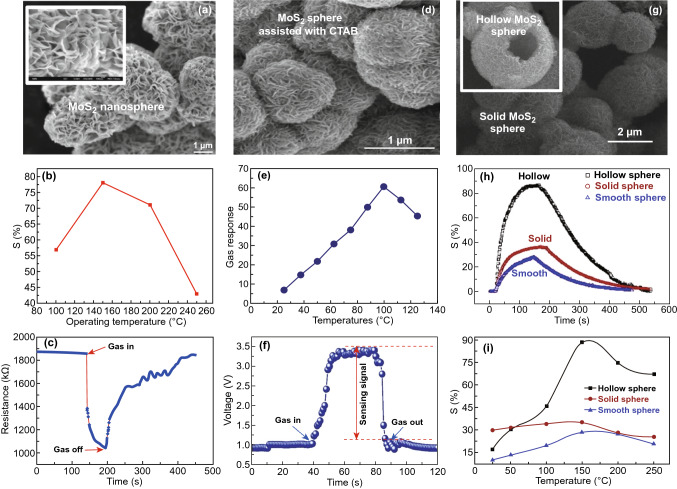
**a** FESEM image of the MoS_2_ nanosphere. **b** Sensor response profile. **c** Transient resistance profile. Reproduced with permission from Ref. [33]. Copyright (2016) from Elsevier. **d** FESEM image of the CTAB-assisted MoS_2_ sphere. **e** Gas response obtained at different temperature range. **f** Time response profile of NO_2_ sensing. Reproduced with permission from Ref. [306]. Copyright (2018) from Elsevier. **g** FESEM image of solid MoS_2_ sphere and hollow sphere. **h** Obtained sensor response for hollow, solid and smooth spheres. **i** Sensor response at different temperatures. The highest sensor response obtained for the hollow spheres. Reproduced with permission from Ref. [307]. Copyright (2019) from Elsevier

Yu et al. adopted the facile hydrothermal method and fabricated the edge-enriched flower-like MoS_2_ spheres [33]. The diameter of the structure estimated through the SEM was 1–2 µm displayed in Fig. 13a. These nanospheres exhibited a large surface area with edge-enriched MoS_2_ flakes. Also, the flakes were interconnected with each other and provided a quick path for the diffusion of gas molecules and charge transfer. The inset of Fig. 13a showed the high magnification FESEM image. These unique structures showed excellent sensor response, cyclability, and selectivity. The 50 ppm NO_2_ concentration is discussed here. The device was operated at different operating temperatures from 100 to 250 °C, displayed in Fig. 13b. The highest sensor response for the 50 ppm NO_2_ concentration was 78% at 150 °C. The resistance versus time profile is shown in the inset of Fig. 13c, confirming the p-type nature of MoS_2_ due to a decrease in the resistance of the sensor device.

Zhang et al. proposed the controlled growth of 3D flower-like MoS_2_ nanospheres assisted with cetyltrimethyl ammonium bromide (CTAB) [306]. CTAB played a crucial role in determining the morphology of the MoS_2_ spheres. The average size of the synthesized nanospheres was 300 nm, displayed in Fig. 13d. This SEM revealed that these MoS_2_ nanospheres were formed due to the bending of the randomly assembled MoS_2_ sheets. These structures provide the path for the diffusion of the gas. The NO_2_ sensing performance was studied in the operating temperature range from RT to 130 °C as shown in Fig. 13e. The highest reported sensor response was 60% observed for the 100 °C temperature. The MoS_2_ nanospheres behaved as the n-type semiconductor. The response and recovery time profile was 15 and 12 s for 50 ppm NO_2_ at 100 °C shown in Fig. 13f.

Li et al. followed a new step and prepared the hollow, solid and smooth MoS_2_ nanospheres by the hydrothermal methods [307]. The hydrothermal process reaction time was maintained at 2-h, 5-h, 18-h in the presence of polyvinyl pyrrolidone (PVP) to synthesize the various morphology MoS_2_ flakes. The polystyrene template (PS) spheres are the platform for the nucleation of MoS_2_ nanosheets. The SEM images of a fully prepared solid sphere and hollow spheres (inset) are shown in Fig. 13g. The 500 ppm NO_2_ concentration is tested in the temperature range from 25 to 250 °C. Hollow spheres have shown the remarkably high sensor response with p-type nature, as shown in Fig. 13h, i. About 2.5-fold enhancement is observed in the hollow spheres compared to solid spheres observed.

Nanospheres improved NO_2_ sensing due to the large surface area of the spheres. The sensing mechanism between the MoS_2_ and NO_2_ is based on the transfer of charge carrier concentration between them. The oxygen gas is adsorbed on the MoS_2_ and introduced p-type doping in MoS_2_. When NO_2_ gas exposed to MoS_2_ spheres, the NO_2_ accepts the electrons from MoS_2_ and gets adsorbed as ${\text{NO}}_{2}^{ - }$ on MoS_2_. Moreover, NO_2_ also reacts with adsorbed ${\text{O}}_{2}^{ - }$ and gets adsorbed as ${\text{NO}}_{2}^{ - }$. The possible reactions of adsorbed oxygen and with NO_2_ are as follows [311]:

8 $$ {\text{O}}_{2} \left( {\text{g}} \right) \to O_{2} \left( {{\text{ads}}} \right) $$

9 $$ {\text{O}}_{2} \left( {{\text{ads}}} \right) + {\text{e}}^{ - } \to {\text{O}}_{2}^{ - } \left( {{\text{ads}}} \right) $$

10 $$ {\text{O}}_{2} \left( {{\text{ads}}} \right) + {\text{e}}^{ - } \to 2{\text{O}}_{2}^{ - } \left( {{\text{ads}}} \right) $$

11 $$ {\text{O}}^{ - } \left( {{\text{ads}}} \right) + {\text{e}}^{ - } \to {\text{O}}_{2}^{ - } \left( {{\text{ads}}} \right) $$

12 $$ {\text{NO}}_{2} + {\text{e}}^{ - } \to {\text{NO}}_{2}^{ - } $$

13 $$ {\text{NO}}_{2} \left( {{\text{gas}}} \right) + {\text{e}}^{ - } \to {\text{NO}}_{2}^{ - } \left( {{\text{ads}}} \right) $$

14 $$ {\text{NO}}_{2} \left( {{\text{gas}}} \right) + {\text{O}}_{2}^{ - } \left( {{\text{ads}}} \right) + 2{\text{e}}^{ - } \to 2{\text{NO}}_{2}^{ - } \left( {{\text{ads}}} \right) + 2{\text{O}}_{2}^{ - } \left( {{\text{ads}}} \right) $$

15 $$ {\text{NO}}_{2}^{ - } \left( {{\text{ads}}} \right) + {\text{O}}_{2}^{ - } \left( {{\text{ads}}} \right) \to 2{\text{O}}^{ - } \left( {{\text{ads}}} \right) + {\text{NO}}_{2} $$

The above discussion clearly shows that different MoS_2_ morphologies could boost the efficiency of gas sensors such as high sensor response, speed (response and recovery time), and selectivity. By choosing the different synthesis modes such as mechanical exfoliation, chemical exfoliation and CVD techniques, various MoS_2_ morphologies can be synthesized ranging from in-plane MoS_2_, flower like MoS_2_, MoS_2_ NWs, vertical MoS_2_ flakes. Different MoS_2_ morphologies like vertical aligned, nanowires, solid and hollow spheres provide the path for the diffusion of gas molecules into the nanostructures so that gas molecules interacts more efficiently. Each morphologies have its own advantage and contributes in improving gas sensing. The MoS_2_ flowers have a high surface area and provide higher adsorption sites for gas molecule adsorption. The hollow microspheres offer a larger surface area (inner and outer surface for molecule adsorption) than the solid spheres. The one-dimensional MoS_2_ NWs provides a combination of high surface area and active sites which will be further increased at moderate temperature.

### Experimental Investigation of Metal Nanoparticle Doping of MoS_2_

In Sect. 4.4, we already discussed various theoretical reports where MoS_2_ was doped with different metal atoms and the advantage of metal doping in MoS_2_ predicted for NO_2_ sensing. Here, we addressed the experimental picture of metal doping in MoS_2_ for NO_2_ sensing. MoS_2_ has a large surface to volume ratio which provides unique opportunities to surface functionalization with metal nanoparticles (NPs) such as Ag, Au, Pt, Pd, and Rh and many more. The incorporation of MoS_2_ surface with metal NPs could be an efficient way to improve the electronic, optical, energy storage and catalytic properties [312–316]. Undoubtedly, functionalizing the MoS_2_ film with metal NPs could open up a new era in the gas-sensing applications.

**Fig. 14:**
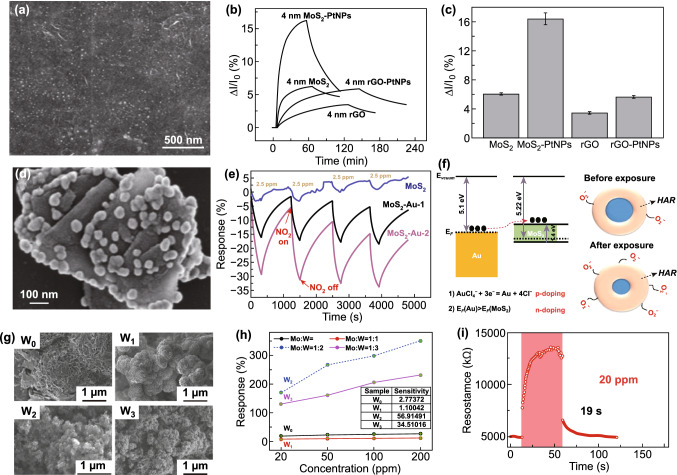
**a** Pt-doped 4-nm-thick MoS_2_. **b** Response of the Pt NP-doped MoS_2_, MoS_2_-rGo and of bare rGO. Pt NP-doped MoS_2_ showed the highest response for the NO_2_ adsorption. **c** Selectivity profile. Reproduced with permission from Ref. [42]. Copyright (2012) Wiley-VCH. **d** Au NP-doped MoS_2_. **e** Response profile with different concentrations of Au decorated MoS_2_ and with UV light exposure. **f** Band alignment of MoS_2_ and Au NPs and mechanism of NO_2_ adsorption. Reproduced with permission from Ref. [121]. Copyright (2018) AIP Publishing. **g** Different concentration W metal loaded MoS_2_. **h** Sensor response profiles of W loaded MoS_2_. **i** Response and recovery profile for 20 ppm NO_2_ exposure. Reproduced with permission from Ref. [320]. Copyright (2020) Elsevier

He et al. used metal nanoparticles to fabricate NO_2_ gas sensor based on MoS_2_ flakes. The 4-nm-thick MoS_2_ film was functionalized with Pt NPs [42]. The FESEM image of Pt-doped MoS_2_ film is shown in Fig. 14a. The comparative sensing performance of Pt NP-doped MoS_2_, rGO-MoS_2_, bare rGO, and bare MoS_2_ is shown in Fig. 14b. The highest sensor response was achieved with Pt-doped MoS_2_. The modulated Schottky barrier height and spillover effect was responsible for enhanced NO_2_ sensing of Pt-doped MoS_2_ [317–319]. The Pt NPs formed nano-Schottky barriers at different places with MoS_2_. Pt extracted the electrons from the MoS_2_ film and introduced p-type doping in MoS_2_. Moreover, due to the spillover effect, the catalytic reactivity of NO_2_ molecules was also increased. Hence, the high sensor response with Pt-doped MoS_2_ was achieved. The selectivity of the Pt-doped MoS_2_ is shown in Fig. 14c.

Zhou et al. decorated Au nanoparticles on MoS_2_ film and performed the NO_2_ detection stability of the MoS_2_-Au composite [121]. The gold NPs of 50 nm diameter formed a strong bond with defects present on the edges. The Au NPs adsorbed at the edges can be seen in Fig. 14d. The NO_2_ sensing ability in the dark and with the UV light exposure was also performed. The MoS_2_ and MoS_2_-Au composites exhibited p-type nature. The full recovery with bare MoS_2_ and Au decorated MoS_2_ in the dark did not be achieve. However, when sensors were illuminated with UV light, fast response with a complete recovery and a three-time greater sensor response was achieved. Figure 14e shows all sensor responses. The band diagram between MoS_2_ and gold NPs are shown in Fig. 14f. It revealed that electrons were transferred from gold NPs to MoS_2_ due to the difference in work function. Au NPs increased MoS_2_ activity and catalytic reactivity [321]. Under UV illumination, charge transfer between the MoS_2_ and Au NPs rapidly increased and led to a fast recovery. The physiosorbed O_2_ and chemically adsorbed O_2_ also produce a hole accumulation (HAL) layer in the MoS_2_ surface similar to the metal oxides. Under NO_2_ exposure, the width of the HAL layer increases and the resistance of MoS_2_-Au decreases.

Liu et al. doped MoS_2_ with different ratios of W metal [320]. The W metal atoms were doped in the following ratio Mo: W: 1:0, 1:1, 1:2, and 1:3 and nominated as W_0_, W_1_, W_2_, and W_3_. The FESEM images of all the four samples are shown in Fig. 14g. The average crystallite size of the W metals was 52, 45, 29, and 32 nm. When NO_2_ gas exposed to W-doped MoS_2_ film, sample W_2_ showed the highest sensor response among all with the fastest response and recovery time.

The undoped MoS_2_ has the numerous number of defects. NO_2_ gas molecules adsorbed on these defective sites through the chemisorption process which leads to strong adsorption between the MoS_2_ and NO_2_ molecules. Hence, NO_2_ desorption is difficult from MoS_2,_ which leads to the sluggish recovery. Metal doping is an efficient way to improve the sensing performance. Here, authors doped MoS_2_ with atoms of W metal which have close radii to Mo atoms. There are no additional defects produced in MoS_2_ due to comparable radii of the Mo and W atoms. Thus the defects in MoS_2_ are significantly suppressed with W metals, and NO_2_ sensing performance is enhanced. The highest sensor response achieved was 56.91% in W_2_ sample, can be seen from Fig. 14h. Interestingly, the response and recovery were the fastest for the sample W_2_. The observed response and recovery time were 24 and 19 s, shown in Fig. 14i.

It is clear from the proposed discussion that metal NPs doping is an efficient way to enhance the gas-sensing performances of MoS_2_ gas sensors. Metal (NPs) doping not only improved the chemical and catalytic reactivity in MoS_2_ but also affected the electronic properties. Metal NPs formed nano-Schottky barriers in different regions of the MoS_2_, which greatly increases the transfer of charges in MoS_2_. Thus, metal (NPs) doping also helps in full recovery of the MoS_2_-based NO_2_ sensors with improved sensor response, selectivity and long term stability. In addition, illuminating the metal (NPs)-doped MoS_2_ sensors could improve the sensing characteristics. However, some more rigorous efforts are still needed to completely explore the effect of light illumination on metal-doped MoS_2_.

### Vacancy-Driven NO_2_ Sensors

**Fig. 15:**
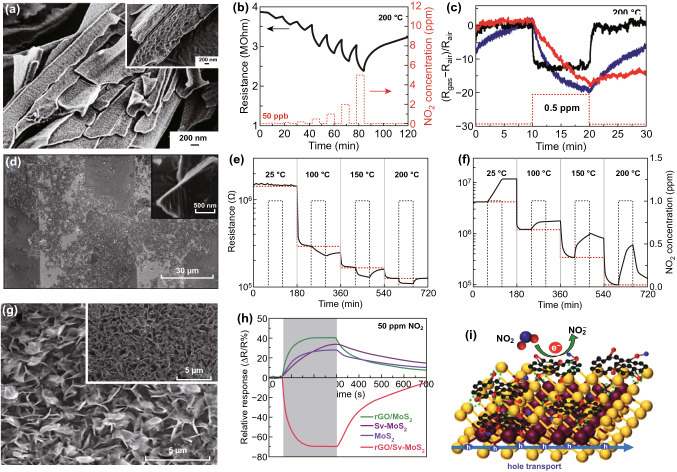
**a** FESEM image of 3D MoS_2_ aerogels. **b** Response of MoS_2_ aerogel with NO_2_ at 200 °C. **c** Device response to NO_2_ at different temperatures. Reproduced with permission from Ref. [24]. Copyright (2017) Wiley-VCH. **d** FESEM image of MoS_2_ flakes distributed on the Pt electrodes. NO_2_ response of the MoS_2_ flakes: **e** annealed at 150 °C showed p-type behavior; **f** annealed at 250 °C showed n-type behavior. Reproduced with permission from Ref. [31]. Copyright (2015) Elsevier. **g** FESEM image of sulfur vacancy-enriched MoS_2_ flakes. **h** Response of 50 ppm NO_2_ with MoS_2_, rGO/MoS_2_, Sv-MoS_2_, rGO/Sv-MoS_2_. rGO deposited Sv-MoS_2_ shown p-type behavior. **i** Mechanism of NO_2_ interaction with rGO deposited Sv-MoS_2_. Reproduced with permission from Ref. [310]. Copyright (2019) IEEE

Vacancies in MoS_2_ played a key role and contributed to increased efficiency in gas sensing. Long et al. synthesized 3D MoS_2_ aerogel by the thermal decomposition technique [24]. A two-step sulfur treatment method was employed to fabricate the NO_2_ gas sensor. Figure 15a and its inset shows surface morphology without treatment and with treatment. The MoS_2_ aerogel became more pours after the sulfur treatment. The MoS_2_ aerogel showed a high sensor response to NO_2_ gas at RT, and a rapid response and full recovery with the sulfur treatment device. The as prepared MoS_2_ aerogels showed a good sensor response. However, due to the strong bonding of NO_2_ with MoS_2_, it suffered from slow response and recovery. The sulfur treatment in the H_2_ ambient produces new sulfur vacancies. The elevated temperature generally removes the S atoms from MoS_2_ and increases the vacancies in sensing film. Figure 15b, c displays the resistance versus time profile for 50 ppb NO_2_ concentration at 200 °C. Furthermore, the response and recovery time were further improved with the temperature attributed to the fast desorption of NO_2_ molecule at high temperature. Donarelli et al. reported the formation of n and p-type MoS_2_ flakes annealed at 250 and 150 °C [31]. The SEM image of MoS_2_ flakes deposited onto the Si_3_N_4_ with Pt electrodes is shown in Fig. 15d. Figure 15e, f shows the relative response of MoS_2_ flakes to 150 and 250 °C. With the electron acceptor nature of NO_2_, the resistance of the MoS_2_ device annealed at 150 °C was decreased while resistance was increased at 250 °C. The device annealed at 150 °C did not respond at RT but a high sensor response was obtained at RT when the device was annealed at 250 °C. Moreover, the sensor showed better sensing performances with 250 °C annealed devices. The n-type and p-type behavior of different devices can be understood in terms of used synthesis method. The NMP was used for the synthesis of MoS_2_. The NMP intercalate in between the MoS_2_ layers at 150 °C. The NMP degraded and introduced the N atom at the S vacancy sites. N atom is an electron acceptor and responsible for p-type behavior [322]. In addition, MoS_2_ surface was partially reduced to MoO_3_ layers and more S and O vacancies were created when MoS_2_ flakes are annealed at 250 °C [323]. The interaction between NO_2_ sensing and n-type MoS_2_ is crucially dependent on the S and O vacancies [324]. Equations 16 and 17 demonstrate the possible reaction mechanism between p-type MoS_2_ and NO_2_.

16 $$ {\text{MoS}}_{2} + {\text{V}}_{{\text{S}}} + {\text{NO}}_{2} + {\text{e}}_{{{\text{CB}}}}^{ - } \to {\text{MoS}}_{2} + ({\text{NO}}_{2}^{ - } + {\text{V}}_{{\text{S}}} ) $$

17 $$ {\text{MoO}}_{3} + {\text{V}}_{{\text{O}}} + {\text{NO}}_{2} + {\text{e}}_{{{\text{CB}}}}^{ - } \to {\text{MoO}}_{3} + ({\text{NO}}_{2}^{ - } + {\text{V}}_{{\text{O}}} ) $$

where $({\text{NO}}_{2}^{ - } + {\text{V}}_{{\text{O}}} )$ and $({\text{NO}}_{2} + {\text{e}}^- )$ are the adsorbed NO_2_ on the oxygen and S vacancies. ${\text{e}}_{{{\text{CB}}}}^{ - }$ is the electron in the conduction band. Hence, NO_2_ interacts with ${\text{e}}_{{{\text{CB}}}}^{ - }$ and leads to a decrease in the conduction band electrons with an increase in the resistance of the electrons.

Kumar et al. annealed the vertical aligned MoS_2_ flakes at 600 °C to obtain vacancy-enriched MoS_2_ flakes. The S atom has low binding energy of (2.12 eV). Hence annealing the MoS_2_ flakes at higher temperatures could be useful to create the S vacancies. Further, the MoS_2_ flakes were decorated with the crumpled rGO. The FESEM image of vertical aligned MoS_2_ flakes is shown in Fig. 15g and rGO decorated flakes showed in the inset of Fig. 15g. The dynamic sensing response of pristine MoS_2_, Sv-MoS_2_, rGO-MoS_2_, and rGO/Sv-MoS_2_ investigated at 50 °C with 50 ppm NO_2_ concentration and showed in Fig. 15h.

The NO_2_ sensor response was 27%, 34%, and 39% for pristine MoS_2_, Sv-MoS_2_, and rGO-MoS_2_, respectively. However, the full recovery and high sensor response of 72% was achieved with rGO/Sv-MoS_2_. The oxygen present in rGO formed strong bonds with S vacancies of MoS_2_ and attracted 0.997 electrons from MoS_2_/rGO. Therefore, there was sufficient transfer of charge between the MoS_2_ and rGO which modified the nature of MoS_2_ from n-type to p-type. S vacancies specifically play a major role in the charge transfer between MoS_2_ and rGO. When NO_2_ molecules were exposed to rGO/Sv-MoS_2_, electrons were depleted from rGO to MoS_2_ and the Fermi level of rGO shifted towards the valence band. Hence, a large number of electrons transferred from the MoS_2_ to rGO. Also, further NO_2_ exposure enhances the holes in MoS_2_ and therefore MoS_2_ behaves as a p-type. The schematic of the proposed mechanism is shown in Fig. 15i.

The role of vacancies in gas sensing has been cleared from the above discussion. The vacancies can change the electronic, optical, and chemical activity of the MoS_2_. The gas molecules interaction at these vacancies sites is governed by the chemisorption process. Thus, the vacancy-enriched MoS_2_ has enhanced NO_2_ sensing performance in terms of sensor response and speed. The vacancies can be tailored through morphology and these vacancies work as the active sites to enhance the gas molecules adsorption. Moreover, the functionalization of vacancies with substitutional atoms can change their electronic nature from n-type to p-type such as N, B, O, and Ni. Another important aspect on the vacancies is the effect of the high temperature annealing of the MoS_2_ film. The high temperature ~ 500–600 °C annealing can generate the more vacancies in MoS_2_ which will be helpful in designing the high-performance NO_2_ sensors based on MoS_2_.

### Light-Assisted NO_2_ Sensors

The MoS_2_ has shown high adsorption energy for NO_2_ molecules at RT. NO_2_ is adsorbed through the chemisorption and physisorption process on the MoS_2_ surface. This high adsorption energy causes difficulty in the full recovery of MoS_2_. MoS_2_ requires additional efforts to remove adsorbed NO_2_ molecules for complete recovery at RT. The one possible solution is to isolate the device temporarily from the toxic environment for complete recovery at high temperatures. However, to develop real time NO_2_ sensor, this method is not feasible. Moreover, it demands necessary engineering efforts which will raise the cost of the sensor and time consuming process. In order to accelerate the desorption rate of NO_2_ molecules from the MoS_2_ surface, researchers used thermal energy to achieve the fast and full recovery of MoS_2_-based NO_2_ sensors. However, there are certain disadvantages of running sensors at elevated temperatures. The speedy recovery is achieved at the cost of the lower sensor response. In addition, it also deteriorates the sensor's long term stability, which raises the complexity and cost of manufacturing sensing devices. Thus, it is not an effective way to run the NO_2_ sensor at high temperatures. The light illumination could be an effective way to enhance the sensing performance of MoS_2_-based sensors while keeping the sensor at RT. The light illumination greatly influences the adsorption, desorption and the adsorption energy. Here, we will focus on the impact of light illumination on the NO_2_ sensing in this section. We have divided the light illumination into three parts UV light illumination, visible light illumination and finally in the NIR illumination.

#### ***Ultraviolet-Activated NO***_***2***_*** Sensor***

**Fig. 16:**
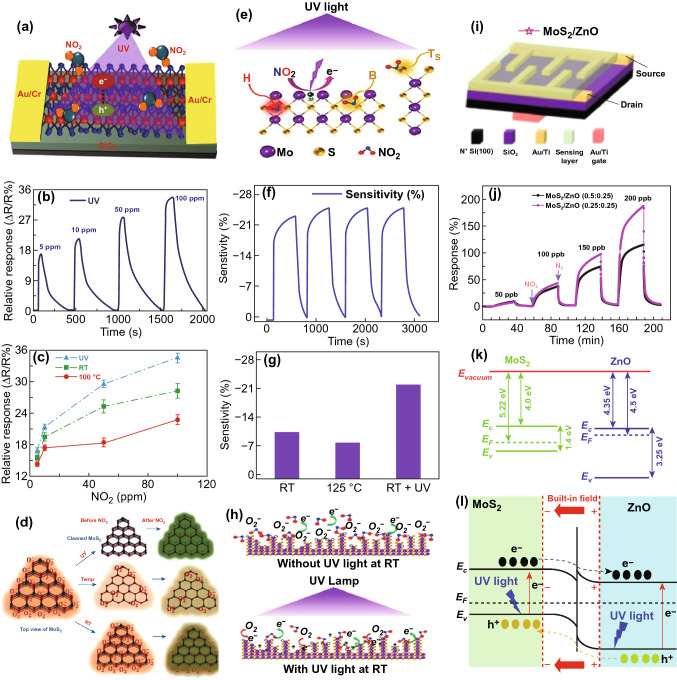
**a** Schematic view of the proposed device under UV illumination. **b** NO_2_ response under UV light, **c** comparative performance under UV at RT, RT, and at higher temperature. **d** Working mechanism. Reproduced with permission from Ref. [119]. Copyright (2017) American Chemical Society. **e** Proposed working mechanism under UV light. **f** Sensor response profile under UV light. **g** Comparative NO_2_ sensing bar profile. **h** Working mechanism under UV illumination. Reproduced with permission from Ref. [120]. Copyright (2018) American Chemical Society. **i** Device schematic for NO_2_ sensing. **j** NO_2_ response with different concentration MoS_2_/ZnO composite device. **k**, **l** Band alignment before the junction and after the formation of contact with NO_2_ exposure. Reproduced with permission from Ref. [325]. Copyright (2018) The Royal Society of Chemistry

Kumar et al. studied the role of the UV light in developing the RT NO_2_ sensor [119]. The CVD grown in-plane MoS_2_ flakes was utilized for NO_2_ gas sensing. The device schematic is shown in Fig. 16a. The NO_2_ sensing was carried out at RT, 100 °C, and with UV illumination at RT (Fig. 16b, c). Among them, the highest sensor response with full recovery was found with the UV light illumination at RT (Fig. 16c). The sensor did not recover fully at RT without UV lighting. The sensor response under tunable UV light intensities from 0.3 to 2 mW cm^−2^ was tested. The sensor response was lowest at 2 mW cm^−2^ and the highest sensor response was recorded at 1.2 mW cm^−2^. The high light intensity allows NO_2_ molecules to desorb easily than their adsorption. Thus, NO_2_ sensor response was lowest at a high light intensity.

Agrawal et al. utilized mixed MoS_2_ flakes for NO_2_ sensing [120]. The NO_2_ sensing at RT, 125 °C, and with UV light illumination at RT was explored. The highest sensor response, fast and full recovery were obtained with UV light illumination at RT. The schematic of the mixed MoS_2_ flakes with possible NO_2_ adsorption sites, the response of mixed MoS_2_ flakes and comparative sensor response under UV light is shown in Fig. 16e–g. The gas-sensing mechanism for both the studies is discussed as follows. The sensing behavior of MoS_2_ flakes is highly dependent on the surface morphology, the number of active sites and notably on the defects in the form of vacancies. The environmental impurities such as oxygen and humidity get adsorbed on these defects. The adsorbed oxygen takes the electrons from the MoS_2_ flakes and introduces p-type doping. At RT without UV light illumination, a high amount of oxygen is adsorbed on the MoS_2_ flakes and a large number of electrons are extracted from MoS_2_ flakes. Owing to the electron acceptor, NO_2_ withdrawn electrons from the MoS_2_. However, the desorption rate is not fast due to the strong bonding of NO_2_ and led to incomplete recovery. Moreover, when thermal energy is added in MoS_2_ from external sources, some oxygen in the MoS_2_ flake is desorbed and more fresh active sites in the form of defects are formed. In addition, thermal energy speeds up the desorption process that causes the sensor response to decrease. The desorption rate of oxygen molecules was highest under UV illumination. UV light illumination generates new electron and hole pairs. The photogenerated holes react with adsorbed oxygen and adsorbed oxygen gets released from the MoS_2_ surface. The UV light illumination creates more fresh active sites. On these fresh active sites, the NO_2_ molecules get adsorbed and increase sensor response. Moreover, when NO_2_ gas turned off, the adsorbed oxygen reacted with the photogenerated electrons and desorbed easily from the MoS_2_ surface. The recovery rate therefore improves under UV lighting. The proposed sensing mechanism for both the reports is shown in Fig. 16d, h.

Zhou et al. fabricated an ultrasensitive, fast UV assisted, RT NO_2_ sensor [325]. The detection limit of the fabricated MoS_2_/ZnO NO_2_ was very low (50 ppb). The n-type ZnO NWs were synthesized using the hydrothermal process, while the ultrasonic method was used to synthesize the p-type MoS_2_. Two types of sensors were fabricated with different composites amount of MoS_2_ and ZnO such as MoS_2_/ZnO (0.5:0.25) and MoS_2_/ZnO (0.25:0.25). The device schematic is shown in Fig. 16i. The bare MoS_2_ device did not show any NO_2_ sensing capability which may be due to the low conductivity of the flakes. However, both devices exhibited significant sensor response under UV exposure. Moreover, devices with equal MoS_2_ and ZnO composites showed better NO_2_ sensing performance under UV light illumination, can be seen from Fig. 16j. The gas-sensing mechanism was proposed based on the band alignment as shown in Fig. 16k, l. MoS_2_ has p-type nature and electrons transferred from the MoS_2_ conduction band to the ZnO conduction band under UV illumination. Thus, the photogenerated charge carriers were segregated efficiently and prevent further recombination.

#### ***Visible Light-assisted NO***_***2***_*** Sensors***

The UV light illumination has evidently proved its significance and its critical role in achieving the fast recoverable NO_2_ sensors for the RT. However, the UV illumination has certain disadvantages as well. Practically, the use of UV light is still a vivid challenge. UV radiation is harmful to human wellbeing. World cancer research agency identified that the continuous use of UV radiation is harmful to humans. Continuous exposure of UV light can cause premature aging of the skin in terms of wrinkles, leathery skin and solar elastosis. UV radiation is therefore particularly harmful to human vision. UV radiation can easily damage the corona of the eyes. The UV rays can significantly affect the immune system. Furthermore, the cost of UV lamps is very high. Therefore, it is essential to study the role of visible light on the gas sensing.

Late et al. studied the role of light exposure in NO_2_ gas sensing. Traditionally, the UV light is the most adopted light source for sensing measurements. However, continuous UV light exposure may degrade the sensing performance of the device and harmful to humans [327]. Thus, the authors used safe green light of 532 nm to perform the NO_2_ gas-sensing measurements.

**Fig. 17:**
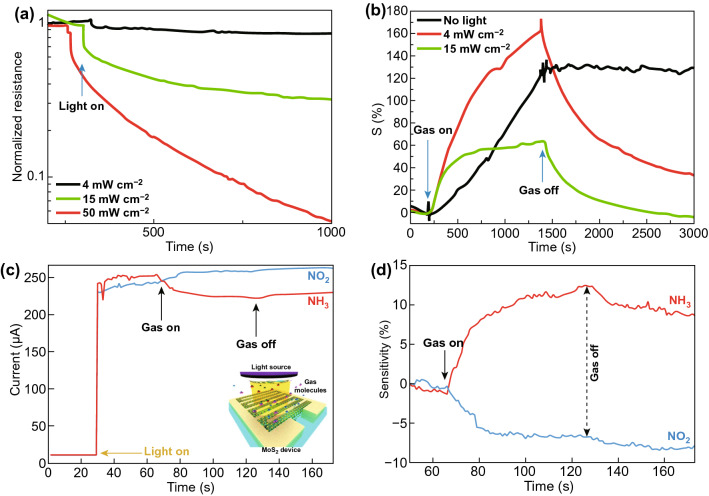
**a** Effect of power density on the resistance. **b** Effect of power density on the NO_2_ response. Reproduced with permission from Ref. [17]. Copyright (2013) American Chemical Society. **c** Detection of the NO_2_ and NH_3_ exposure with 650 nm wavelength exposure. **d** Change in the sensor response under light illumination. Reproduced with permission from Ref. [326]. Copyright (2015) American Chemical Society

The irradiated green light has tunable power densities from 4 to 50 mW cm^−2^. The highest resistance change has been observed with higher incident power, which is attributed to the higher number of photogenerated electrons and holes with higher incident light power. The change in the resistance with incident light power density is shown in Fig. 17a. With light illumination, the desorption rate of NO_2_ gas molecule is relatively high in comparison with the adsorption rate. Moreover, a small fraction of electrons reacts with NO_2_ gas due to the high power density of incident light. Therefore, the NO_2_ sensor response is reduced with a high incident power density as shown in Fig. 17b. The full recovery is obtained with green light illumination. Similarly, Cho et al. synthesized atomic layered MoS_2_ and illuminated device with 650 nm red light [326]. The schematic of the device is shown in the inset of Fig. 17c. The photogenerated current increased rapidly when the red light was turned on after 30 s. NO_2_ gas was turned on after 60 s. The current increased further implying the p-type characteristic of the MoS_2_ flakes. The calculated sensor response with red light illumination is shown in Fig. 17d.

#### ***Near-Infrared (NIR)-Assisted NO***_***2***_*** Sensor***

**Fig. 18:**
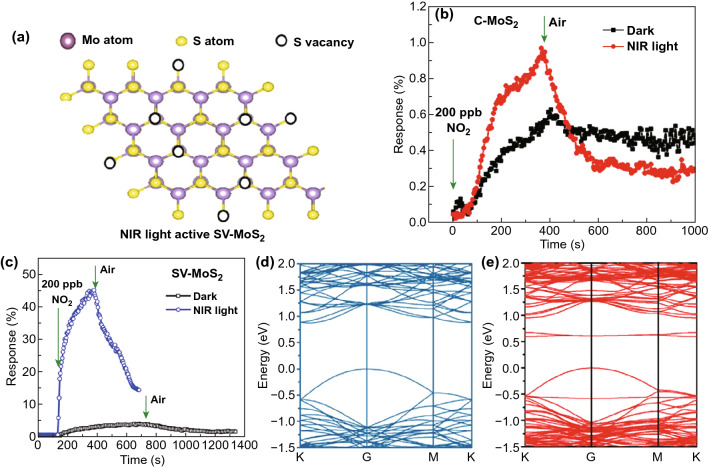
**a** Schematic of NIR light-activated sulfur vacancy-enriched MoS_2_ (Sv-MoS_2_). **b** Response of C-MoS_2_ in dark and NIR with NO_2_ exposure. **c** Response of Sv-MoS_2_ in dark and NIR with NO_2_ exposure. Band structure of **d** C-MoS_2_ (blue) and **e** Sv-MoS_2_ (red). Reproduced with permission from Ref. [123]. Copyright (2019) American Chemical Society. (Color figure online)

Xia et al. recently used NIR light to develop sensitive fast NO_2_ sensor with sulfur vacancy-enriched MoS_2_ flakes [123]. The conventional MoS_2_ (C-MoS_2_) and sulfur vacancy-enriched MoS_2_ (S-MoS_2_) flakes were synthesized by the traditional microwave-hydrothermal method. The sulfur vacancies were investigated by the electron paramagnetic resonance (EPR), XPS, and XRD. The schematic structure of C-MoS_2_ and Sv-MoS_2_ is shown in Fig. 18a. Further, the absorption spectroscopy has been performed for both the C-MoS_2_ and Sv-MoS_2_ which revealed the high absorption of NIR light by Sv-MoS_2_. The NO_2_ sensing ability of the C-MoS_2_ and Sv-MoS_2_ is shown in Fig. 18b, c. Interestingly, the observed sensor response with Sv-MoS_2_ was high in the presence and in the absence of NIR light. The presence of S vacancies modulated the band structure of MoS_2_ flakes and generated three additional localized states in MoS_2_ bandgap, i.e., two unoccupied states at 0.63 eV below the conduction band and one shallow state near the valence band (Fig. 18d, e). Both additional states narrow down the MoS_2_ bandgap in contrast with the pure MoS_2_ bandgap. Hence, Sv-MoS_2_ showed a high NIR photoresponse.

The S vacancies quickly reduced the Gibbs free energy of adsorbed gas molecules and increased the electron transfer rate from MoS_2_ to NO_2_. The Sv-MoS_2_, therefore provides enhanced sensing efficiency not only in the dark but also with NIR lighting. The gas sensing performances of light driven MoS_2_-based NO_2_ sensors summarized in Table 4.

**Table 4:** Summary of the light-driven NO_2_ sensor of MoS_2_

Sensing film	Method	Electrodes	Def.	Device	Conc. (ppm)	*P*/*λ* (mW/cm^2^/nm)	T (°C)	S (%)	Res time	Rec. time	References
Multi MoS_2_	CVD	Au/Cr	$\frac{{\left( {R_{\text{gas}} - R_{\text{air}} } \right)}}{{R_{\text{air}} }}$	Resistor	100	1.2/365	RT + UV	35.16	29	350	[119]
Mix MoS_2_	CVD	Au/Cr	$\frac{{\left( {R_{\text{gas}} - R_{\text{air}} } \right)}}{{R_{\text{air}} }}$	Resistor	10	3/365	RT + UV	21.78	6.1	146.5	[120]
MoS_2_/ZnO	Hydrothermal	Au/Ti	$\frac{{\left( {R_{\text{gas}} - R_{\text{air}} } \right)}}{{R_{\text{air}} }}$	FET	50	1/365	RT + UV	188	60	60	[325]
MoS_2_	CVD	Pt	$\frac{{R_{\text{gas}} }}{{R_{\text{air}} }}$	Resistor	10	0.29/365	RT + UV	25.3	–	63.9	[328]
ZnO/MoS_2_	Ultrasonication	ITO	$\frac{{\left( {R_{\text{gas}} - R_{\text{air}} } \right)}}{{R_{\text{air}} }}$	Resistor	10	360	RT + UV	293	258	72	[329]
1L MoS_2_	CVD	Cr/Pd/Au	$\frac{{\left( {R_{\text{gas}} - R_{\text{air}} } \right)}}{{R_{\text{air}} }}$	Resistor	0.4	4/625	RT + 625	670	16	65	[122]

These studies revealed that under light illumination gas-sensing performance of MoS_2_ is critically affected. Light illumination is a promising approach to enhance the sensor response of MoS_2_ in comparison with providing thermal activation. The electrons and holes pairs generated due to light illumination provide a sufficient number of charge carriers to increase the gas-sensing response of the MoS_2_ sensors. Traditionally, UV light is the most verified technique to enhance the sensor response of gas sensors and also with MoS_2_. UV illumination provides better treatment of adsorbed ambient oxygen than thermal energy. UV illumination significantly cleans environmental oxygen from the MoS_2_ surface without any structural loss than the thermal energy. But, long term exposure of UV is not good for living cells.

Furthermore, the integration of MoS_2_ with NO_2_ sensitive materials could be helpful in developing ultrasensitive NO_2_ sensors at RT with light. MoS_2_-Heterojunctions rapidly separate the generated electron and holes pairs due to light and NO_2_ exposure, which will improve the gas-sensing performance.

MoS_2_ has a high absorption coefficient in the visible region of spectrum of spectrum. Thus, a large number of electrons generate in MoS_2_ in visible region and NO_2_ has high number of electron available to withdraw from MoS_2_ surface. However, with UV light, the number of generated electrons holes pairs are not so high. So, utilizing the visible spectra in gas sensing could be a better and safe approach to fabricate the high-performance gas sensors. To further utilize the NIR spectra, some engineering efforts may be needed to enhance the absorption of MoS_2_ in NIR. Use of NIR light sources will reduce the high cost of the sensors in comparison with UV and visible light sources.

## MoS_2_-Heterostructure NO_2_ Sensor

Advancement in MoS_2_ gas sensors can be achieved by forming the heterostructures. The production of single or few layer MoS_2_ is considered not an easy approach and limits the high throughput of gas sensor. Ambiguity in the gas-sensing mechanism of MoS_2_ with NO_2_ gas has also been a topic of debate. Integration of MoS_2_ with other materials such as graphene derivative, metal oxides and carbon materials create heterostructure at the junction. The formation of heterostructure affects the gas-sensing properties in both positive and negative aspects. Forming a heterojunction can improve the intrinsic electronic properties of MoS_2_ that tends to improve the sensor response and recovery time. However, the integration of heterostructure also puts a bit of complexity in the gas-sensing mechanism. Here, in this section, we tend to summarize the advancement in the material of different dimensions with MoS_2_ equipped gas sensor over time.

Despite showing high sensor response by few layer MoS_2_-based TFT sensor, their low conductivity limits the performance of device [34, 218]. The high surface to volume ratio of graphene and its derivatives opened up possibilities of hybrid gas sensors, where graphene and its derivative provided better electrical conductivity to the device. Theoretical calculations done using DFT have expounded that pollutant gases, like NO_2_, NO, and SO_2_, firmly interact with MoS_2_ surfaces. Numerous experimental confirmations of these theoretical results have been reported. A three-layer-grown MoS_2_-based resistive sensor showed a NO_2_ detection limit of 120 ppb in dark conditions [326].

In order to improve the sensing behavior, the blending of MoS_2_ with graphene nanosheet was adopted [42, 330, 331]. To enhance the sensor response and selectivity to NO_2_ more, a composite of reduced graphene oxide (rGO) and MoS_2_ was prepared [332]. The p-type nature of rGO, due to oxygen and water doping and n-type nature of MoS_2_ make p–n junction. MoS_2_ provided selectivity and sensibility, while rGO had provided betterment in electronic properties. Zhou et al. also fabricated rGO/MoS_2_ gas sensor for NO_2_ detection [333]. The fabricated composite structure showed 200% enhanced performance than the bare rGO sensor. The device showed sensing response of 59.8% towards 2 ppm NO_2_ at 60 °C.

**Fig. 19:**
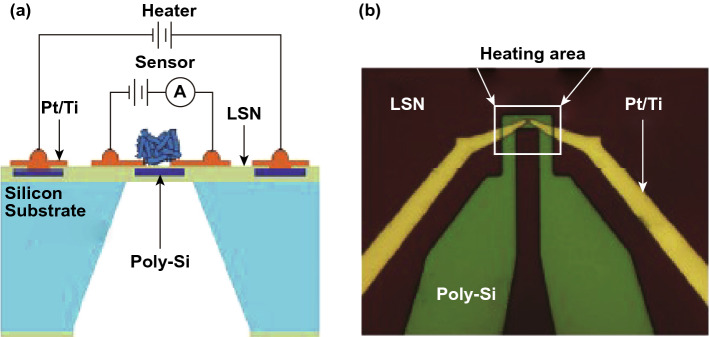
**a** Schematic diagram of NO_2_ gas sensor with microheater sensor. **b** Optical image of poly-silicon microheater with Pt/Ti electrodes. Reproduced with permission from Ref. [118]. Copyright (2016) Wiley-VCH

On the contrary, Long et al., fabricated gas sensor using MoS_2_/graphene hybrid aerogel for NO_2_ detection [118]. The MoS_2_/graphene-based sensor showed ultralow detection limit of 50 ppb NO_2_. The hybrid was integrated over low power micro heater for temperature-dependent gas detection measurement and on heating at 200 °C sensor show improved recovery and response time of less than 1 min compared to RT measurements. The schematic and optical image of the device with microheater is shown in Fig. 19.

Many other works have been reported where the sensing performance was improvised by using graphene over MoS_2_ [334, 335]. Despite the good selectivity of the MoS_2_/rGO sensor, the MoS_2_ has serious issues like agglomeration on the substrate. Therefore, the fabrication of MoS_2_ gas sensor with enhanced sensing activity was a challenge. CdS was used as sensing material and assumed to provide good electron transfers between heterostructure [336].

Tabata et al., fabricated gate tunable MoS_2_/graphene NO_2_ sensor [29]. To understand the role of only heterojunction, the other gas-sensing parts were passivated by the gas barrier layer. Poly methyl methacrylate (PMMA) was used as gas barrier layer. The device showed the strong dependencies on the type of bias (forward or reverse) and back gate voltage. With an increase in reverse bias and negative gate voltage device showed better performance. Jung et al. also fabricated the flexible gas sensor by transferring the MoS_2_/rGO on the PET substrate that showed optical transmittance ~ 93% [337]. The flexible MoS_2_/rGO showed good performance at a bending radius of 14 mm and detection as low as 0.15 ppm of NO_2_.

On the contrary, Ikram et al. used a thin layer of In(OH)_3_ on the MoS_2_ nanosheets to improve the performance of NO_2_ gas sensor [338]. The presence of point and line defects in MoS_2_/In(OH)_3_ improves the electrical conductivity and provides the accessibility of active sites for target gas. The ease of fabrication of MoS_2_/MoO_3_ composites in one step has also grabbed attention and the sensor showed a remarkable sensor response of ~ 33.6% with complete recovery to 10 ppm NO_2_ at RT [339]. The sensor response of 2D materials to the surrounding critically affect the long-term reliability of the sensing device. Therefore, Shi et al. fabricated a layered device using black phosphorus (BP) as the top gate, Boron nitride as a dielectric layer and MoS_2_ as conduction channel [340]. The gas adsorption ability of BP makes it a gas-sensing material and BN isolates the conduction channel of MoS_2_ from ambient. The multilayered gas sensor showed a detection limit of 3.3 ppb to NO_2_. The SnS_2_ nanosheets were also used to fabricate the sensor due to their high adsorption sites availability and showed response 22.3 times higher than pristine SnS_2_ sensor [341].

**Fig. 20:**
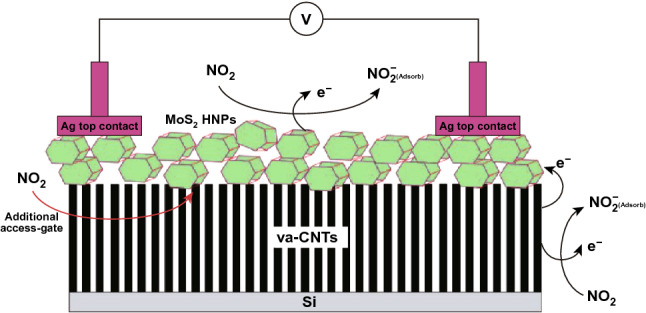
Schematic of MoS_2_ deposited over CNT-based device. Reproduced with permission from Ref. [342]. Copyright (2017) Wiley-VCH

Apart from the integration of 2D nanostructure with MoS_2_, integration of 1D also offers enhancement in gas-sensing properties. Deokar et al. fabricated CNT/MoS_2_-based hybrid NO_2_ gas sensor [342]. Hexagonal shaped MoS_2_ nanoplates were grown on vertical aligned CNTs. Few tens (25, 50, 100) of ppm to hundreds (25, 100) of ppb of NO_2_ at RT was monitored. An illustration of the gas-sensing mechanism of 2D/1D heterostructure is depicted in Fig. 20.

Zhao et al. fabricated a hybrid of MoS_2_/porous Si nanowire [343]. The MoS_2_ nanosheets were grown by sulfurization of Mo thin film deposited using DC magnetic sputtering. The hybrid device showed better performance than bare MoS_2_ and porous Si NW with low detection concentration of 1 ppm. Keeping the success of MoS_2_ and porous Si NW in attention, ZnO nanowires were also used in forming the heterostructure [344]. MoS_2_ was grown by the same sulfurization of Mo thin films deposited by DC magnetic sputtering. The MoS_2_ on ZnO NW showed an excellent sensor response, recovery, repeatability and selectivity up to low detection of 200 ppb. MoS_2_, that naturally act as n-type semiconductor forms heterostructure with n-type ZnO NW and charge interfacial charge separation takes place. The electron in the CB of MoS_2_ flows to the CB of ZnO NWs till their Fermi level gets aligned. The type of heterostructure (type I and type II) can be decided by the band gap and the work function of the two materials. Similarly, other reports were also reported where ZnO nanowires were used with p-type MoS_2_ nanosheets to improve the sensors performance [345].

**Fig. 21:**
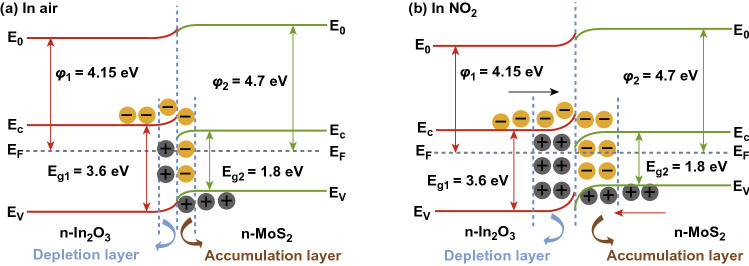
Energy band diagram of In_2_O_3_/MoS_2_ heterojunction in **a** air and **b** NO_2_ ambient. Reproduced with permission from Ref. [346]. Copyright (2019) Elsevier

On the contrary to NW and CNTs, hollow tubes have also been considered and effective 1D nanostructure for enhancing the gas-sensing properties. Yang et al. fabricated NO_2_ gas-sensing device using In_2_O_3_ hollow tube and MoS_2_ nanoparticles [346]. The In_2_O_3_/MoS_2_ composite synthesized by layer by layer technology. Both n-type metal oxides nanostructure and n-type MoS_2_ form a heterostructure. Depending upon the band gap and work function, the majority carrier flows from CB of MoS_2_ to CB of metal oxide (MO) nanostructure and vice versa till their Fermi level gets aligned. When the sensor is exposed in the air ambient, the oxygen molecules capture electrons from MoS_2_ and MO nanostructure and forms ${\text{O}}_{2}^{ - }$ ions. An energy band diagram that explains the sensing mechanism in the air and in NO_2_ ambient is shown in Fig. 21. Once the sensor is exposed into NO_2_ ambient, the NO_2_ molecules capture electrons from the sensing layer and adsorbed ${\text{O}}_{2}^{ - }$, hence increase the device resistance.

When the sensor is again released in the air ambient, the electron trapped by NO_2_ is again released to CB sensing materials leading to the restoration of device resistance. In band diagram terminology, when the sensor is exposed to the NO_2_, the electrophilic nature of NO_2_ reduces the carrier concentration in the depletion region resulting an increase in the resistance. Therefore, the quality of the interface between the MoS_2_ and MO nanostructure greatly affects the sensing properties.

Among different heterojunctions, 2D/0D offers improved gas-sensing properties (sensor response, recovery time, and improved time) due to the enhanced penetration and diffusion of gas molecules. The first 2D/0D hybrid heterostructure was fabricated and demonstrated by Han et al. for gas NO_2_ gas sensing. Han and co-workers fabricated 2D/0D heterojunction using MoS_2_ nano-sheets and ZnO NP that exhibited sensor response of 3050% for 5 ppm NO_2_ and long term stability of 10 weeks at RT [347].

**Fig. 22:**
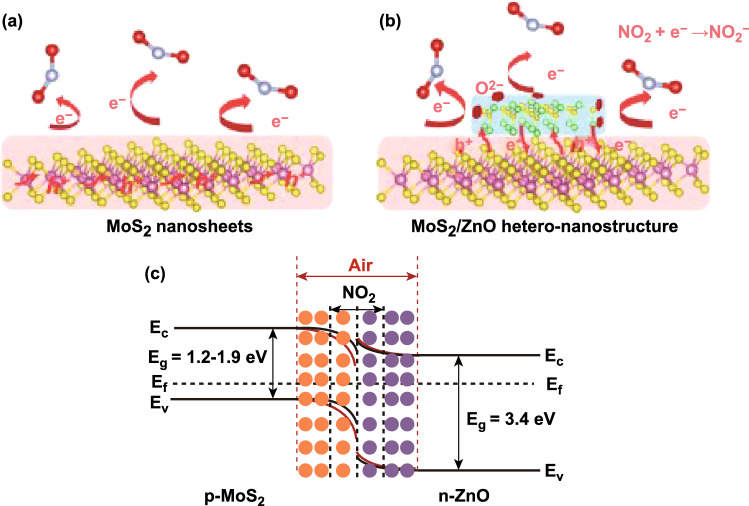
Schematic of sensing mechanism of **a** pure MoS_2_ nanosheets and **b** hybrid mechanism. **c** Band diagram of hybrid 2D/0D heterojunction gas sensor in air ambient and in the NO_2_ ambient. Reproduced with permission from Ref. [347]. Copyright (2015) American Chemical Society

The gas-sensing mechanism of 2D/0D heterostructure is explained with the help of Fig. 22. The defects on the surface of as deposited p-type MoS_2_ act as active sites for gas sensing. NO_2_ molecules accept electrons from MoS_2_ and change the electronic properties of the sensor. The integration of metal oxide 0D structure (n-type) over p-type forms a p–n junction followed by the formation of the depletion layer. The electron and hole diffusion keep happening from n-type to p-type and p-type to n-type, respectively, till their fermi levels get aligned. A built-in electric field balances the flow of majority carrier. Therefore, in the air, the MoS_2_/ ZnO junction shows poor conductivity due to the formation of potential barrier. When the sensor is brought in the ambience of NO_2_, the NO_2_ molecules take electrons from metal oxide nanoparticle and the equilibrium of electron and hole is broken. The extra holes that were counter balanced by the electrons taken up by the NO_2_ molecule migrate to the MoS_2_. Therefore, during adsorption of NO_2_, holes are accumulated on the MoS_2_ surface and the width of depletion layer decreases that leads to the increase in conductivity of MoS_2_/ZnO heterostructure. The increase in the conductivity of heterostructure enhances gas-sensing properties.

Another metal oxide nanoparticle that was used for the hybrid MoS_2_ sensor was SnO_2_ NPs. The MoS_2_/SnO_2_ NO-based sensor showed a response from 18.7 to 5 ppm NO_2_ [348]. Similarly, Xin et al. fabricated device using PbS quantum dots [349].

The MoS_2_/PbS QDs showed sensing performance better than bare MoS_2_. The response showed by the hybrid device was 50 times higher than pure MoS_2_ at 100 ppm with recovery ratio of around 99%. In another report, Ag nanoparticle was used for surface modification of the Fe_2_O_3_/MoS_2_ heterojunction. The modified sensor showed response to 5 ppm NO_2_ as high as 202.2% which was 11Times higher than the bare MoS_2_ device [27]. On the contrary, n-type MoS_2_ was also used for device fabrication. The n-type-CdTe quantum dots also used on n-type MoS_2_ nanoworms to enhance the sensing performance [350]. The device showed an excellent response of 40% over pristine MoS_2_. The band bending of n-type MoS_2_ and n-type 0D is shown in Fig. 22c. We summarized the gas-sensing performances of various MoS_2_ heterostructures in Table 5.

**Table 5:** Summary of various MoS_2_ heterostructures NO_2_ sensors

Sensing film	Electrodes	Conc. (ppm)	Def	T (°C)	S (%)	Res time (s)	Rec time (S)	References
Graphene/MoS_2_	Au	50	$\frac{{\left( {R_{\text{gas}} - R_{\text{air}} } \right)}}{{R_{\text{air}} }}$	RT	83	–	–	[332]
rGO/MoS_2_	Au	2	$\frac{{\left( {R_{\text{gas}} - R_{\text{air}} } \right)}}{{R_{\text{air}} }}$	60	59.8	–	–	[333]
rGO/MoS_2_	Au	3	$\frac{{R_{\text{gas}} }}{{R_{\text{air}} }}$	160	1.23	8	20	[351]
Graphene/MoS_2_	Pt/Ti	0.5	$\frac{{\left( {R_{\text{gas}} - R_{\text{air}} } \right)}}{{R_{\text{air}} }}$	200	10	21.6	29.4	[118]
MoS_2_/graphene	Au	10	$\frac{{\left( {I_{\text{air}} - I_{\text{gas}} } \right)}}{{I_{\text{air}} }}$	200	69	0.7	0.9	[334]
Graphene/MoS_2_	–	500	$\frac{{\left( {R_{\text{gas}} - R_{\text{air}} } \right)}}{{R_{\text{air}} }}$	RT	61	22	35	[335]
rGO/MoS_2_/CdS	NA	0.2	$\frac{{\left( {R_{\text{gas}} - R_{\text{air}} } \right)}}{{R_{\text{air}} }}$	75	27.4	25	34	[336]
MoS_2_/In(OH)_3_	NA	100	$\frac{{R_{\text{air}} }}{{R_{\text{gas}} }}$	RT	15.4	1.6	NA	[338]
MoS_2_/MoO_3_	Au	10	$\frac{{\left( {R_{\text{gas}} - R_{\text{air}} } \right)}}{{R_{\text{air}} }}$	RT	33.5	19	182	[339]
SnS_2_/MoS_2_	Au	100	$\frac{{R_{\text{air}} }}{{R_{\text{gas}} }}$	RT	25.9	2	28.2	[341]
MoS_2_/CNT	Ag	50	$\frac{{R_{\text{gas}} }}{{R_{\text{air}} }}$	RT	12.6	–	–	[342]
MoS_2_/p-Si NWs	Ag	50	$\frac{{\left( {R_{\text{gas}} - R_{\text{air}} } \right)}}{{R_{\text{air}} }}$	RT	28.4	–	–	[343]
MoS_2_/ZnO NW	Silver	50	$\frac{{\left( {R_{\text{gas}} - R_{\text{air}} } \right)}}{{R_{\text{air}} }}$	200	29.4	300	–	[344]
ZnO NW/MoS_2_	Au/Ti	50 ppb	$\frac{{\left( {R_{\text{gas}} - R_{\text{air}} } \right)}}{{R_{\text{air}} }}$	60	63	–	–	[345]
MoS_2_/In_2_O_3_ NT	–	50	$\frac{{R_{\text{air}} }}{{R_{\text{gas}} }}$	RT	209	–	–	[346]
MoS_2_/ZnO	Au	5	$\frac{{\left( {I_{\text{gas}} - I_{\text{air}} } \right)}}{{I_{\text{air}} }}$	RT	3050	40	1000	[347]
MoS_2_/SnO_2_ NPs	Au/Ti	5	$\frac{{R_{\text{gas}} }}{{R_{\text{air}} }}$	RT	18.7	74	NA	[348]
Ag-Fe_2_O_3_/MoS_2_	Ag-Pd	5	$\frac{{\left( {R_{\text{gas}} - R_{\text{air}} } \right)}}{{R_{\text{air}} }}$	120	202.2	81	355	[27]
CdTe/MoS_2_	Ag	10	$\frac{{\left( {R_{\text{gas}} - R_{\text{air}} } \right)}}{{R_{\text{air}} }}$	RT	40	16	114	[350]
MoS_2_/SnO_2_	Au	10	$\frac{{\left( {R_{\text{gas}} - R_{\text{air}} } \right)}}{{R_{\text{air}} }}$	RT	28	408	162	[352]

## Challenges and Future Perspectives

MoS_2_ has shown immense gas-sensing capacity without any doubt and also shown great performance in the detection of various gas molecules like NO_2_, NH_3_, H_2_, H_2_S etc. However, some major issues still have to be overcome in order to boost the efficiency of MoS_2_ gas sensors. There are several crucial challenges to be overcome to build high-performance MoS_2_-based NO_2_ sensors. Gas-sensing performance depends on certain important parameters including sensor response, recovery time, selectivity, and stability. MoS_2_ is extremely susceptible to different gases, and its conductivity varies dramatically under exposure to these gases. A small exposure of any gas to MoS_2_ will notably change sensor response. Thus, the detection of target gas molecules is critical by MoS_2_.

Theoretically, NO_2_ and NH_3_ both have almost identical adsorption energy with similar adsorption sites. However, NO_2_ has an electron acceptor nature, while NH_3_ has an electron donor nature. The synthesis of such morphology which is highly selective for NO_2_ molecules is therefore advantageous and can increase NO_2_ selectivity. MoS_2_ has a high surface to volume ratio, so it is useful to functionalize MoS_2_ flakes with NO_2_ capturing agents to improve NO_2_ adsorption on MoS_2_ flakes.

Another benefit of MoS_2_ is the effective control on morphology. Morphology influences the gas diffusion in the sensing film. The role of different morphologies in detecting NO_2_ has been already studied in detail with various conventional metal oxides, including ZnO, SnO_2_ and in TiO_2_ etc. So, there is plenty of space for NO_2_ gas detection by morphology-driven sensor. In addition, NO_2_ molecule adsorption on MoS_2_ depends greatly on the position, so any effort to increase the NO_2_ adsorption sites can not only enhance the sensor response but also boost the selectivity ability. The RT recovery is yet another big challenge for MoS_2_-based NO_2_ gas sensors. MoS_2_ has strong adsorption energy with NO_2_ gas molecules. Currently, bare MoS_2_-based NO_2_ sensors have experienced incomplete recovery at RT. RT thermal energy is not capable to desorb the adsorb NO_2_ gas. This demands the operation of sensors at elevated temperature from RT. However, this will happen at the cost of reduce sensor response performance of the sensor. So thermal treatment for achieving full recovery is not feasible. Recently the light-assisted recovery of the gas sensors is open a new promising way to develop RT gas sensors. Light illumination not only helps in the recovery of the sensors but it also enhances the 3S performance (low response and recovery time and sensor response). Bare MoS_2_-based sensors have improved RT recovery under UV light illumination so far.

Very few attempts have been made in recent years to use residual spectrum (visible, NIR, and higher region). Thus, an intensive approach is still required to explore the wavelength-dependent NO_2_ gas-sensing response and to explore light induced carrier generation and adsorption of NO_2_ molecules.

Apart from the sensor response and recovery time, fast response of the gas sensor is also an essential parameter. Each sensor's response time depends on how rapidly the gas molecules reacted to sensing film and change their respective parameter. Till now, the reported response time of NO_2_ molecules detection by MoS_2_ is in few seconds. So, developing NO_2_ sensors which can respond in few milliseconds or microseconds is still challenging. The strategy to improve the ultrafast sensors relies primarily on the interaction between gas molecules and MoS_2_ and the charge transfer in MoS_2_. The fast separation of the charge carriers can be improved by forming MoS_2_ heterostructures sensing devices.

In addition, a proper attention is needed to pay on metal electrodes, which collect generated charges. The metal contacts has played a vital role with MoS_2_ in gas sensing. Identification of high-performance metal contacts is the perquisite to utilizing the full performance of the MoS_2_-based NO_2_ sensors. An improved theoretical and experimental efforts with profound insight and understanding is also needed, which will contribute to the development of high-performance NO_2_ sensors. Several routes to develop high-performance electrical contact should identify.

The standard air quality guidelines, according to WHO for NO_2_ exposure are 82 ppb for an hour and 410 ppb for a year. Exposure to NO_2_ for a long time above that level causes health problems. The recorded lower detection limit for MoS_2_-based NO_2_ sensor has been in the ppb. Thus, a lot of effort is needed to develop ultrasensitive NO_2_ sensors, which is a crucial task. It is important to find NO_2_ sensitive materials that can be easily integrated with MoS_2_, and can identify the lower concentration of NO_2_ easily. Furthermore, such materials should also fasten the transfer of charges for rapid sensor response. Recently, the use of spectroscopic techniques such as laser sources and electrical shields have attracted the attention of the scientific community due to their NO_2_ trace detection ability. NO_2_ molecules have the absorption spectrum in visible region so it offers a great chance to electronic exciton in the visible region of NO_2_ molecules. The use of spectroscopic techniques for trace detection of NO_2_ with MoS_2_ sensors could be a new approach.

Light-assisted NO_2_ sensing has attracted the scientific community over the last two years. Metal NP-doped MoS_2_ sensors have already proved their importance in gas sensing. Surface plasma resonance (SPR) characteristics of MoS_2_ may be a new approach to developing gas sensor-based on MoS_2_. New experimental efforts should devote to harnessing the potential of plasmonic in gas sensing. SPR can stimulate the interface of MoS_2_ and metal and alter the index refractive. A good choice of metal NPs and appropriate wavelengths will be helpful in designing the high-performance NO_2_ sensors.

The MoS_2_-based NO_2_ sensors are basically chemiresistance sensors in which the change in conductance of MoS_2_ film is the parameter. The conductivity of film is significantly influenced by the presence of the environment such as traces of various chemicals, moisture, humidity, corrosion due to toxic vapors, residual charges. These all drastically reduce the reliability, stability and repeatability of gas sensors. Efforts are required to increase the stability of the sensing devices.

## Conclusions

In this review, we summarized the various theoretical and experimental strategies employed to fabricate MoS_2_-based NO_2_ gas sensors. We critically discussed the advantages of utilizing the 2D MoS_2_ in developing NO_2_ sensors. We briefly discussed the noble properties of MoS_2_ and established MoS_2_ as a potential candidate for the gas sensing. The inherent nonzero bandgap, high carrier mobility, fast charge transport, high reactivity, presence of favorable adsorption sites, large surface to volume ratio, and optical properties make MoS_2_ amenable for gas molecule adsorption. Both theoretical and experimental studies have confirmed that NO_2_ adsorption in MoS_2_ is controlled by the charge transfer process. NO_2_ behaves as a strong oxidizing agent and depletes the electrons from MoS_2_. Theoretical studies revealed NO_2_ adsorption in MoS_2_ is position dependent. The 2H and 1T MoS_2_ have their importance in NO_2_ gas sensing. Although most of the work to fabricate MoS_2_-based NO_2_ sensor have been carried out with 2H-MoS_2_ phase, but the 1T MoS_2_ phase is emerged as the potential candidate for NO_2_ detection. The aforementioned hypothesis has been verified theoretically and must be taken into account in experiments as well. Theoretical and experimental studies confirmed that the defective MoS_2_ have higher interaction and have high NO_2_ detection ability than the pristine MoS_2_. Furthermore, metal doping at the vacancy sites is an alternative way to develop highly sensitive, fast response and RT-recoverable NO_2_ sensors. Different MoS_2_ morphologies have different number of NO_2_ adsorption sites. Thus, NO_2_ sensing performance of MoS_2_ could be further improved and determined by morphology.

**Fig. 23:**
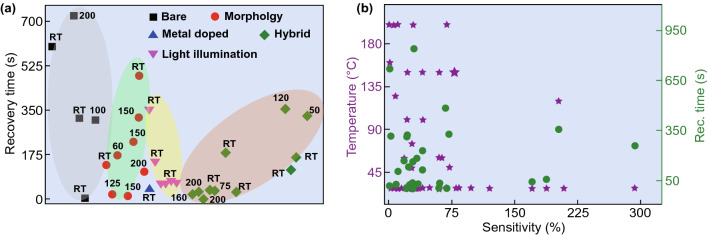
**a** Summary of the recovery time obtained through various strategies in MoS_2_. Bare MoS_2_-based NO_2_ sensors have highest recovery time followed by the morphology-driven MoS_2_. The light-assisted NO_2_ sensors have the lowest recovery time and can operate easily at RT. MoS_2_ heterostructure-based sensors have mixed recovery time with different operating temperatures. **b** MoS_2_-based sensors can operate easily at low temperatures and have low recovery time. Data presented was taken from Refs. [17, 24, 27, 31–36, 118–122, 214, 303–310, 320, 325, 328, 329, 333–336, 338–352]

The formation of MoS_2_ heterostructures can significantly affect the NO_2_ sensing performances. MoS_2_ heterostructures rapidly separate the charges and could be helpful in developing fast response and recover gas sensors. Among all, light-assisted NO_2_ sensors have paved a new path to achieve fast RT-recoverable NO_2_ sensors. Finally, we graphically presented the gas-sensing characteristics such as recovery time, temperature, and sensitivity obtained from various reports in Fig. 23 to have an easy catch over the progress. In Fig. 23a, we have summarized the recovery time obtained through various strategies. Many bare MoS_2_ NO_2_ sensors either have incomplete recovery, or need to operate at high temperature for full recovery. Hence, we have few data points. The data revealed that NO_2_ has high adsorption energy with MoS_2_ at RT. Due to high adsorption energy, bare MoS_2_ NO_2_ sensors are suffered from incomplete or long recovery time at RT. The morphology controlled MoS_2_ sensors have a good recovery but in a moderate temperature range. External thermal energy is needed to recover the MoS_2_ sensors. MoS_2_ heterostructures-based sensors have the mixed recovery time with different operating temperature depending on their partner materials and charge transfer mechanism. It is also observed that MoS_2_ heterostructures NO_2_ sensors have the comparatively less recovery time than the bare MoS_2_ and morphology-driven NO_2_ sensors due to faster separation of charges at RT and moderate temperature. Interestingly, light-assisted NO_2_ sensors have the lowest recovery time with RT operation. Thus, light illumination has played a significant role in improving NO_2_ sensors at RT. Photogenerated electrons and holes pairs crucially help in the desorption of the adsorbed NO_2_ molecules. In Fig. 23b, we have concluded the various reports and summarize the gas-sensing factors. Graphical representation revealed MoS_2_-based NO_2_ sensors have a clear advantage over the traditional sensors in terms of temperature, cost and power. The statistics presented in the Fig. 23b has confirmed that NO_2_ sensors based on MoS_2_ can fill the performance gap shown in the Fig. 3. With traditional metal oxide sensors, we need high operating temperature up to 500 °C while MoS_2_ sensors can easily operate at low temperature range with high sensor response and selectivity with low recovery time.
